# Interaction between host G3BP and viral nucleocapsid protein regulates SARS-CoV-2 replication and pathogenicity

**DOI:** 10.1016/j.celrep.2024.113965

**Published:** 2024-03-15

**Authors:** Zemin Yang, Bryan A. Johnson, Victoria A. Meliopoulos, Xiaohui Ju, Peipei Zhang, Michael P. Hughes, Jinjun Wu, Kaitlin P. Koreski, Jemma E. Clary, Ti-Cheng Chang, Gang Wu, Jeff Hixon, Jay Duffner, Kathy Wong, Rene Lemieux, Kumari G. Lokugamage, R. Elias Alvarado, Patricia A. Crocquet-Valdes, David H. Walker, Kenneth S. Plante, Jessica A. Plante, Scott C. Weaver, Hong Joo Kim, Rachel Meyers, Stacey Schultz-Cherry, Qiang Ding, Vineet D. Menachery, J. Paul Taylor

**Affiliations:** 1 Department of Cell and Molecular Biology, St. Jude Children’s Research Hospital, Memphis, TN, USA; 2 Integrated Biomedical Sciences Program, University of Tennessee Health Science Center, Memphis, TN, USA; 3 Department of Microbiology and Immunology, University of Texas Medical Branch, Galveston, TX, USA; 4 Institute for Human Infection and Immunity, University of Texas Medical Branch, Galveston, TX, USA; 5 Center for Tropical Diseases, University of Texas Medical Branch, Galveston, TX, USA; 6 Department of Infectious Diseases, St. Jude Children’s Research Hospital, Memphis, TN, USA; 7 School of Medicine, Tsinghua University, Beijing, China; 8 Center for Applied Bioinformatics, St. Jude Children’s Research Hospital, Memphis, TN, USA; 9 Faze Medicines, Cambridge, MA, USA; 10 Department of Pathology, University of Texas Medical Branch, Galveston, TX, USA; 11 World Reference Center for Emerging Viruses and Arboviruses, University of Texas Medical Branch, Galveston, TX, USA; 12 Howard Hughes Medical Institute, Chevy Chase, MD, USA; 13 These authors contributed equally; 14 Lead contact

## Abstract

G3BP1/2 are paralogous proteins that promote stress granule formation in response to cellular stresses, including viral infection. The nucleocapsid (N) protein of severe acute respiratory syndrome coronavirus 2 (SARS-CoV-2) inhibits stress granule assembly and interacts with G3BP1/2 via an ITFG motif, including residue F17, in the N protein. Prior studies examining the impact of the G3PB1-N interaction on SARS-CoV-2 replication have produced inconsistent findings, and the role of this interaction in pathogenesis is unknown. Here, we use structural and biochemical analyses to define the residues required for G3BP1-N interaction and structure-guided mutagenesis to selectively disrupt this interaction. We find that N-F17A mutation causes highly specific loss of interaction with G3BP1/2. SARS-CoV-2 N-F17A fails to inhibit stress granule assembly in cells, has decreased viral replication, and causes decreased pathology *in vivo*. Further mechanistic studies indicate that the N-F17-mediated G3BP1-N interaction promotes infection by limiting sequestration of viral genomic RNA (gRNA) into stress granules.

## In brief

Yang et al. characterize the interaction between G3BP1 and SARS-CoV-2 N protein at the single-residue level. They show that a point mutation (N-F17A) selectively disrupts G3BP1-N interaction and reduces viral replication and pathology *in vivo*, demonstrating that this interaction promotes infection by limiting sequestration of viral gRNA into stress granules.

## Graphical Abstract

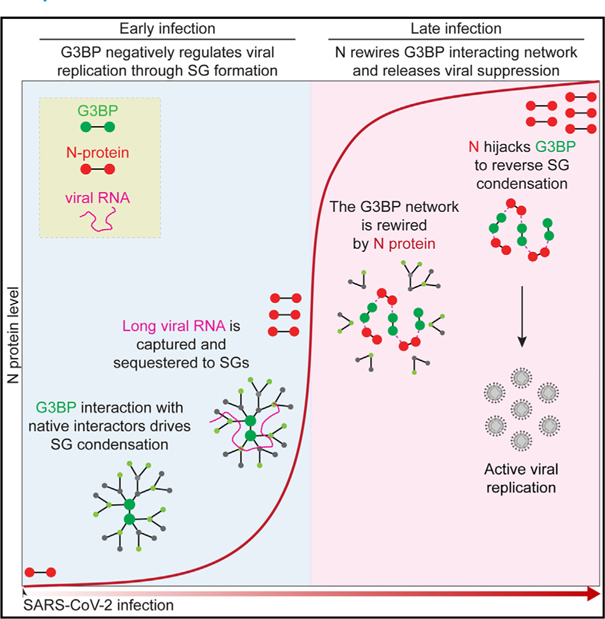

## INTRODUCTION

Severe acute respiratory syndrome coronavirus 2 (SARS-CoV-2), the virus responsible for the COVID-19 pandemic, uses viral proteins to manipulate and control host processes. Among the virus’ four structural proteins, the nucleocapsid (N) protein is primarily responsible for compacting viral genomic RNA (gRNA) inside the virion, likely by promoting condensation of gRNA into ribonucleoprotein (RNP) complexes via phase separation.^1,2^ In addition to this primary function, the N protein has been implicated in several other activities, including viral RNA replication,^3^ subgenomic RNA transcription,^3–5^ and suppression of innate immune responses.^6^ Given its multiple roles, it is critical to understand how the N protein modulates host cells to facilitate SARS-CoV-2 infection. Notably, expression of the SARS-CoV-2 N protein is dependent on the stage of infection; levels of N protein are low in early infection and rise dramatically as infection proceeds, eventually becoming the most abundant viral protein in infected cells.^7–13^

Recent work suggests that SARS-CoV-2 N plays a role in the suppression of stress granules (SGs), which are cytoplasmic RNP condensates that form in response to cellular stresses, including viral infection. The mechanistic connection between the N protein and inhibition of SG formation appears to center on the paralogous RNA-binding proteins G3BP1 and G3BP2, which are prominent host interactors of the N protein and key components of the SG response.^9,14–19^ G3BP1 is an abundant cytoplasmic protein that surveils its milieu for a rise in the concentration of uncoated RNAs, such as occurs during stress responses; following this rise, G3BP1 facilitates condensation of RNAs and proteins to form G3BP1-dependent condensates such as SGs.^20^

Whereas SGs are generally thought to promote antiviral responses, the specific role of G3BP1 in viral infection has been more challenging to define. For example, in many types of viral infection,^21–36^ viruses neutralize G3BP1 activity and inhibit SG assembly to promote viral replication.^37^ Various strategies have evolved to accomplish this antagonism, including cleavage of the G3BP1 protein^21–27^ and/or the production of a protein that directly interacts with a key binding pocket in G3BP1,^28–32^ thereby inhibiting its ability to drive SG assembly. On the other hand, many of these same viruses, including chikungunya virus,^28^ Semliki Forest virus (SFV),^31^ noroviruses,^34^ Zika virus,^35,36^ and hepatitis C virus,^38^ appear to co-opt the condensation properties of G3BP1 to promote viral replication.

SGs are reported to be scant or absent in SARS-CoV-2-infected cells despite the presence of double-stranded RNA, which is capable of inducing SG formation through activation of EIF2AK2 (also known as PKR, protein kinase R).^39–42^ Both SARS-CoV-2 infection and ectopic N expression inhibit SG formation in response to exogenous stimuli.^17,39–45^ However, the relationship between the N protein and G3BP1-dependent SG formation during viral infection *in vivo* is less clear. Indeed, inhibition or loss of G3BP1 has been reported to promote replication in some contexts and cell types^41,42^ but decrease replication in others.^46,47^ Beyond the limitations of interrogating viral pathogenesis in cell culture, prior studies relied on genetic depletion or deletion of G3BP1,^41,42,46,47^ which is confounded by the pleiotropic consequences of loss of function of G3BP1, including compensatory changes in the expression of other SG proteins.

In this study, we sought to clarify the role of the G3BP1-N interaction in SARS-CoV-2 replication and pathogenesis. Following structure-guided mutagenesis of G3BP1 and N to selectively and reciprocally disrupt their interaction, we investigated the impact of disrupting this interaction *in vitro* and *in vivo*. We dissected the structural basis for the G3BP1-N interaction, demonstrating that N-F17A ablates interaction with G3BP1/2 without disruption of other interactors. SARS-CoV-2 with the F17A mutation failed to disrupt SG assembly, showed attenuated viral replication *in vitro*, and caused reduced disease *in vivo*. Mechanistically, we show that sequestration of long viral RNAs by G3BP1-dependent condensates contributes to antiviral defense and that the G3BP1-N interaction circumvents this antiviral response by suppressing G3BP1-dependent condensation of viral gRNA.

## RESULTS

### SARS-CoV-2 N protein directly interacts with G3BP1

**Figure 1.**
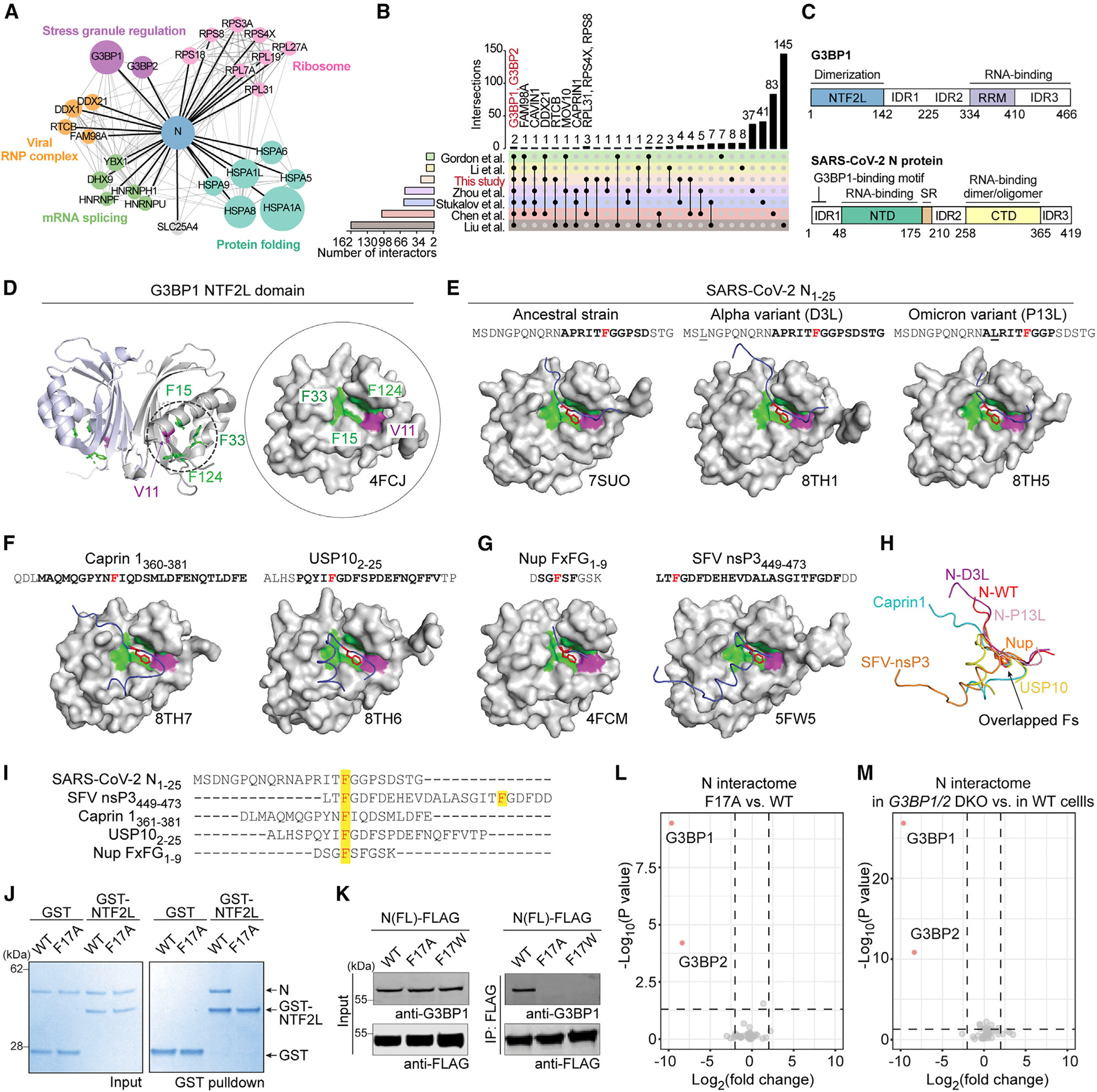
SARS-CoV-2 N protein interacts with G3BP1 via N-F17 (A) N protein interactome as defined by mass spectrometry of HEK293T cells expressing GFP-tagged N protein. All 26 high-confidence interactors are shown, grouped by cellular function. (B) UpSet plot comparing N interactomes identified in this and six previous studies. (C) Domain organization of G3BP1 and N protein. IDR, intrinsically disordered region; RRM, RNA recognition motif; NTD, N-terminal domain; CTD, C-terminal domain. Residues 1–25 of N protein are highlighted as the G3BP1 binding region. (D) Structure of G3BP1 NTF2L with V11, F15, F33, and F124 highlighted. Side chains from these residues create a hydrophobic pocket for protein binding. (E–G) Crystal structures of G3BP1 NTF2L in complex with the indicated peptides. PDB IDs are indicated below the relevant structures; peptide paths are shown in blue. Side chains of relevant phenylalanine residues (N-F17, caprin 1 F372, USP10 F10, Nup-FxFG F4, nsP3 F451) are shown in red. Corresponding residues are shown in red in the aa sequence. Mutated residues are underlined, and bold font indicates aas traceable in the crystal structures. In (G), SFV nsP3 F468 also inserted its side chain into the NTF2L binding pocket (not shown). (H) Overlay of the paths of seven NTF2L-interacting peptides as extracted from crystal structures indicates the variation in paths of the backbone of bound peptides. The orientation of the phenylalanines inserted into NTF2L is conserved across all binding partners. (I) Aa sequence of NTF2L-binding peptides; highlights indicate inserted phenylalanines. (J) GST pull-down of purified GST-NTF2L with N-WT and N-F17A. Input (left) and bound proteins (right) were visualized by Coomassie blue staining. </p/>(K) HEK293T cells were transfected with FLAG-tagged N-WT, N-F17A, or N-F17W. Cell extracts were captured with magnetic beads conjugated with FLAG antibody for immunoprecipitation, and bound proteins were analyzed by immunoblot. (L) Comparison of N-WT versus N-F17A interactomes from HEK293T cells identified G3BP1/2 as the only interactors that were significantly decreased (red) in theN-F17A interactome; all remaining interactors were unchanged (gray). p < 0.05 and fold change of 2 were used as cutoffs. (M) Comparison of N-WT interactomes in G3BP1/2 DKO cells versus parental HEK293T cells identified *G3BP1/2* as the only interactors that were significantly decreased (red) in *G3BP1/2* DKO cells; all remaining interactors were unchanged (gray). p < 0.05 and fold change of 2 were used as cutoffs. See also Figures S1 and S2 and Tables S1 and S3.

Numerous analyses across diverse cell types, systems, and experimental approaches have identified G3BP1/2 as the most prominent host interactors of the N protein of SARS-CoV-2.^9,14–19^ Consistent with these studies, we found G3BP1/2 among the top hits in the N protein interactome in our independent mass spectrometry analysis of HEK293T cells expressing GFP-tagged N protein (Figures 1A and 1B; Table S1). Of the 26 high-confidence interactors, six were identified as functioning in protein folding (*HSPA1A, HSPA1L, HSPA5, HSPA6, HSPA8*, and *HSPA9*), one was identified as being involved in mitochondrial ADP/ATP transport (*SLC25A4*), and the remaining 19 were classified as RNPs involved in various aspects of RNA metabolism, including RNA granule regulation, mRNA splicing, and translation (Figure 1A). Our analysis of six published N protein interactomes,^9,14–19^ combined with our own dataset, revealed that G3BP1/2 were the only proteins consistently identified in all seven studies (Figure 1B).

G3BP1/2 are paralogous proteins that share a domain architecture consisting of an N-terminal NTF2-like (NTF2L) domain that underlies protein homodimerization, two intrinsically disordered regions in the center of the protein (IDR1 and IDR2), and a C-terminal RNA-binding domain composed of a folded RNA recognition motif in tandem with an arginine-glycine-rich IDR3 (Figure 1C). The SARS-CoV-2 N protein has two folded domains: an N-terminal RNA-binding domain and a C-terminal domain that has dimerization/oligomerization and RNA binding capacities.^48–54^ These two folded domains are flanked by three IDRs (IDR1, IDR2, and IDR3) (Figure 1C).^55,56^

We next examined whether the G3BP1-N protein interaction was direct, and if so, which domains mediated this interaction. To this end, we used *in vitro* pull-down assays with purified N (His-small ubiquitin-like modifier [SUMO]-tagged) and G3BP1 (glutathione S-transferase [GST]-tagged) proteins. We found that His-SUMO-N and GST-G3BP1 could each pull down the other protein, indicating a direct interaction (Figure S1A). To test whether this interaction was mediated by the G3BP1 NTF2L domain, we repeated these experiments using purified GST-NTF2L as well as full-length GST-G3BP1 ΔNTF2L. The NTF2L domain alone was able to bind the N protein, whereas the protein lacking NTF2L failed to do so, indicating that the NTF2L domain is necessary and sufficient for interaction with the N protein (Figure S1A).

To identify the G3BP1 binding site within the N protein, we generated a series of N protein truncations with sequential domain deletions (Figure S1B). We found that deletion of N-IDR1 (amino acids [aas] 1–47) was sufficient to abolish the interaction of the N protein with endogenous G3BP1, whereas deletion of IDR2, IDR3, or the C-terminal domain had relatively little effect (Figures S1B and S1C). We then mapped the G3BP1 binding site using serial deletions within IDR1, finding that the first 25 aas were sufficient for a robust interaction with G3BP1 (Figure S1D). These results demonstrate a direct G3BP1-N interaction that requires the NTF2L domain of G3BP1 and the first 25 aas of the N protein, consistent with previous studies showing that N interacts with G3BP1/2 via an ITFG motif at aas 15–18.^43,44,46^

### N protein and G3BP1 interactors bind to G3BP1 NTF2L in a mutually exclusive manner

We next examined the effect of the G3BP1-N interaction on the normal G3BP1 host interaction network. The NTF2L domain harbors the binding site for several types of proteins, including host proteins that regulate SG assembly (e.g., caprin 1^57^ and USP10^58^) and viral proteins (e.g., non-structural protein 3 [nsP3] of alphaviruses^59–61^). Several of these partners bind G3BP1 in a mutually exclusive manner. For instance, caprin 1 and USP10 show mutually exclusive binding to NTF2L, with antagonistic effects on SG formation.^62^ To determine whether these interactors compete with the N protein for binding to NTF2L, we synthesized three peptides derived from the G3BP1-interacting motifs of caprin 1 (aas 361–381), USP10 (aas 1–25), and nsP3 (aas 449–473) from SFV (Figure S1E). We then used an *in vitro* GST pull-down assay with purified GST-NTF2L and His-SUMO-N proteins and added increasing concentrations of caprin 1, USP10, or nsP3 peptides. All three peptides competed against the NTF2L-N interaction in a dose-dependent manner, with the nsP3–25mer peptide showing the most effective competition for binding to the N protein (Figure S1F). We then tested for competitive binding in living cells by expressing nsP3–25-mer-mCherry and G3BP1-GFP *in G3BP1/2* double knockout (DKO) cells. We found that the G3BP1-N interaction was dramatically reduced in the presence of nsP3–25-mer (Figure S1G), demonstrating that this mutually exclusive binding can also occur in living cells.

### A common docking mode: N and other G3BP1 interactors insert a phenylalanine side chain into the center of an NTF2L hydrophobic pocket

The mutually exclusive binding by the N protein and other G3BP1 interactors suggested that these proteins may bind to the same site on NTF2L. However, the primary aa sequences of G3BP1-interacting motifs vary considerably and lack a conserved motif (Figure 1I). Thus, we used crystallography to examine whether a shared interaction mode might exist at the three-dimensional level.

Published structures of G3BP1/2 have revealed that the NTF2L domain consists of a five-stranded antiparallel β sheet and three α helices (Figure 1D; PDB: 4FCJ).^63^ Two NTF2L molecules form a homodimer by the face-to-face stacking of two β sheets. On the outer surface of each dimer, two α helices, with side chains of residues V11, F15, F33, and F124 (Figure 1D, green and purple), create a hydrophobic pocket for protein binding.^63^ This pocket is responsible for the vast majority of G3BP1-protein interactions involved in the formation of a condensation network.^20^

The crystal structure of the G3BP1 NTF2L domain in complex with the first 25 aas of the N protein (N-WT_1–25_) has been reported (PDB: 7SUO).^64^ In this structure, the side chain of N-F17 (Figure 1E, red) was inserted into the center of the NTF2L pocket and interacted with surrounding NTF2L residues. To compare this NTF2L-N interaction with other NTF2L interactors, we obtained the crystal structures of G3BP1 NTF2L in complex with peptides derived from two host proteins, caprin 1_360–381_ (PDB: 8TH7) and USP10_2–25_ (PDB: 8TH6) (Figure 1F). Similar to SARS-CoV-2 N protein and consistent with published structures,^57,58^ both peptides inserted one phenylalanine (F372 in caprin 1, F10 in USP10) into the same NTF2L pocket. The use of this pocket is also evident in structures of NTF2L interacting with an FxFG peptide derived from host nucleoporin (Nup) protein (PDB: 4FCM)^63^ and with the nsP3 protein of SFV (PDB: 5FW5),^60^ where phenylalanines (F4 in Nup FxFG peptide, F451 and F468 in nsP3) were inserted into this pocket (Figure 1G).

Two SARS-CoV-2 variants of concern,^65–67^ Alpha and all extant Omicron variants, have acquired mutations within the first 25 aas of the N protein, raising the possibility that these mutations might alter the G3BP1-N interaction. To test this, we determined the crystal structures of G3BP1 NTF2L in complex with N-D3L_1–25_ (Alpha variant mutation^68^) (PDB: 8TH1) and N-P13L_1–25_ (Omicron variant mutation^69^) (PDB: 8TH5) (Figure 1E). Comparing these structures with NTF2L bound to N-WT_1–25_, we found that all three N peptides bound to the same NTF2L pocket by inserting the F17 side chain (Figure 1E, red) into the center of this pocket. N-D3L could not be visualized, indicating that this residue is located some distance from the NTF2L-N interaction surface (Figure 1E). N-P13L, although in close proximity to the interacting interface, was not essential for the interaction (Figure 1E). N-P13L slightly altered the orientation of the peptide in proximity to the NTF2L interacting site, raising the possibility of alterations in binding between NTF2L and N-P13L, a prediction we pursue below. Nonetheless, we found that the G3BP1-N interaction was essentially unaltered even after the emergence of SARS-CoV-2 variants. Indeed, after overlaying the structures of all NTF2L-interacting peptides, we found that the side chain of the inserted phenylalanines of different peptides adopted a nearly identical conformation (Figures 1H and 1I).

We next tested these structural observations in cells by mutating relevant phenylalanine residues in peptides derived from NTF2L interactors and assessing the ability of these peptides to bind G3BP1. Consistent with our structural predictions, substituting phenylalanines with alanines in caprin 1 (F372A), USP10 (F10A), nsP3 (F451A/F468A), or SARS-CoV-2 N (F17A) abolished their interaction with endogenous G3BP1 in HEK293T cells (Figure S1H). We also tested the effect of F17A and F17W substitutions in full-length N protein, finding that both F17A and F17W abolished the G3BP1-N interaction when tested with purified proteins (Figure 1J) and in cells (Figure 1K).

### N-F17A specifically abolishes interaction with G3BP1/2

To examine the consequences of the SARS-CoV-2 N-F17A mutation on the interaction of the N protein with other host proteins, we compared the interactome of N-wild type (WT) and N-F17A in HEK293T cells by GFP tag-based affinity purification and mass spectrometry. Remarkably, the only host proteins that showed significant changes across duplicate experiments were G3BP1/2 (Figure 1L; Table S1). We also defined the interactome of the N-WT protein in HEK293T *G3BP1/2* DKO cells and found that G3BP1/2 were again the only significant changes observed for the N interactome between WT and *G3BP1/2* DKO cells (Figure 1M; Table S1). These results suggest that N-F17 is specifically utilized for interaction with G3BP1/2 in host cells and that G3BP1/2 do not act as intermediates to recruit other interactors to the N protein. These insights created a unique opportunity to specifically interrogate the functional significance of the G3BP1-N interaction in a physiological context without the pleiotropic effects attendant to knockout (KO) or knockdown studies.

### N-F17Adoes notalter *invitro* phase separation behavior of N protein

During viral infections, the N protein engages in both homotypic interactions with other N proteins and heterotypic interactions with RNA molecules. These interactions underlie the ability of the N protein to undergo liquid-liquid phase separation.^1^ To test whether F17 affects these interactions, we assessed the ability of purified recombinant N-WT and N-F17A to undergo phase separation *in vitro*. We found that phase separation of N proteins triggered by the addition of a crowding agent (Figure S2A) or RNA (Figure S2B) was unaltered between N-WT and N-F17A, indicating that N-F17A does not disrupt heterotypic interactions with RNA or homotypic interactions with other N proteins. We further assessed the ability of N-WT and N-F17A to bind RNA in cells using cross-linking and immunoprecipitation, finding that N-WT and N-F17A bind similarly to cellular RNAs (Figure S2C).

### G3BP1-N interaction is conserved among all SARS-CoV-2 variants

**Figure 2.**
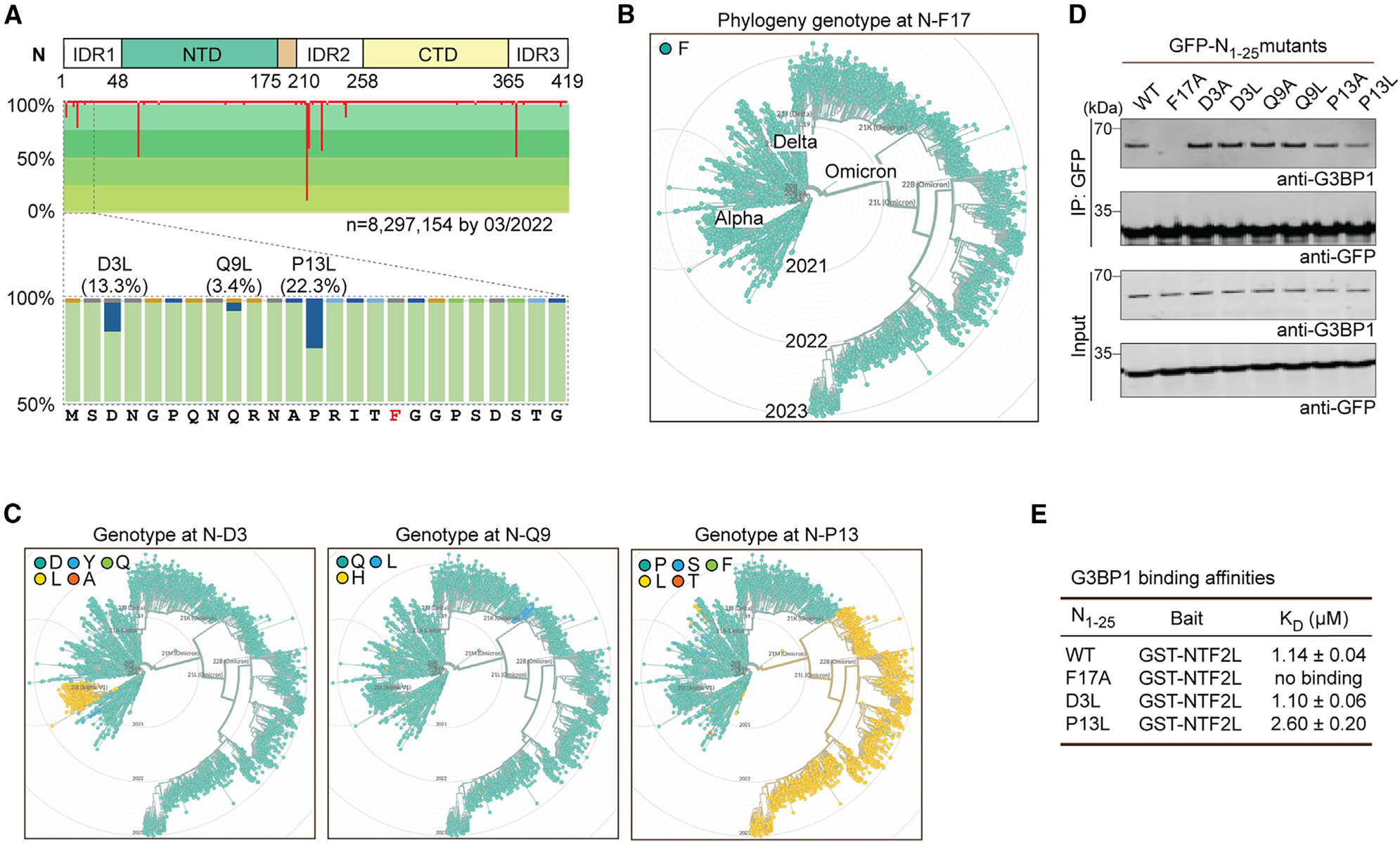
G3BP1-N interaction is conserved among SARS-CoV-2 variants (A) Aa variations of N protein among SARS-CoV-2 genomes deposited in GISAID. Sites with aa replacements are highlighted in red, with line length indicating aa variations (%) at that site. The conservation of each aa is represented by shades of green. F17 (red font) showed no aa variations. (B and C) Phylogeny genotypes of N protein at F17 (B), D3, Q9, and P13 (C) are shown in chronological order from 2019 to 2023. (D) HEK293T cells were transfected with the indicated GFP-tagged N_1–25_ mutations. Cell extracts were captured with magnetic beads conjugated with GFP antibody for immunoprecipitation, and bound proteins were analyzed by immunoblot. Representative images from three replicates are shown. (E) Results from the SPR assay, showing binding affinities of the indicated N protein peptides with purified GST-NTF2L *in vitro*. K_D_ values were derived from a global fit of all replicates ± SD.

To determine whether the G3BP1-N protein interaction is conserved among SARS-CoV-2 variants, we analyzed the aa variations of the N protein among 8,297,154 viral genomes deposited in the Global Initiative on Sharing All Influenza Data (GISAID) by March 2022.^70^ Most aas within the protein were highly conserved (Figure 2A). F17 showed no aa variations among all viral genomes (Figure 2A) and was conserved in all lineages, including transient sublineages that appeared and declined over the course of the pandemic (Figure 2B). For the other aas within aas 1–25, most aas were also highly conserved. Exceptions here were three aa replacements, D3L, Q9L, and P13L, two of which we examined above using crystallography, which were observed in 13.3%, 3.4%, and 22.3% of all SARS-CoV-2 genomes, respectively (Figure 2A). D3L appeared specifically in the Alpha variant, whereas Q9L was observed in a sublineage of the Delta variant. Interestingly, P13L appeared sporadically among early variants in 2020 (lineages 20A, 20B, 20C, and 20D by Nextstrain),^71^ disappeared in Alpha, Gamma, and Delta variants, and then re-appeared in the Omicron variant and its sublineages (Figure 2C).

To determine whether the G3BP1-N interaction was affected by these mutations, we expressed N protein with mutations at these residues (D3A, D3L; Q9A, Q9L; P13A, P13L) in HEK293T cells and tested their interaction with endogenous G3BP1 by co-immunoprecipitation (coIP). All mutants retained the ability to bind G3BP1 (Figure 2D), consistent with our earlier crystal structure data (Figure 1E). Interestingly, N-P13L and N-P13A showed slightly decreased interaction with G3BP1 (Figure 2D). We confirmed this observation using surface plasmon resonance (SPR), which revealed a lower binding affinity of N_1–25_-P13L peptide (K_D_ = 2.6 μM) with purified GST-NTF2L protein *in vitro* compared with N_1–25_-WT (K_D_ = 1.14 μM) and N_1–25_-D3L (K_D_ = 1.10 μM) peptides (Figure 2E). These data demonstrate the conservation of the G3BP1-N interaction across all SARSCoV-2 variants, suggesting that this interaction may be of significance for the virus.

### G3BP1-N interaction specifically involves V11 and F124 of G3BP1

**Figure 3.**
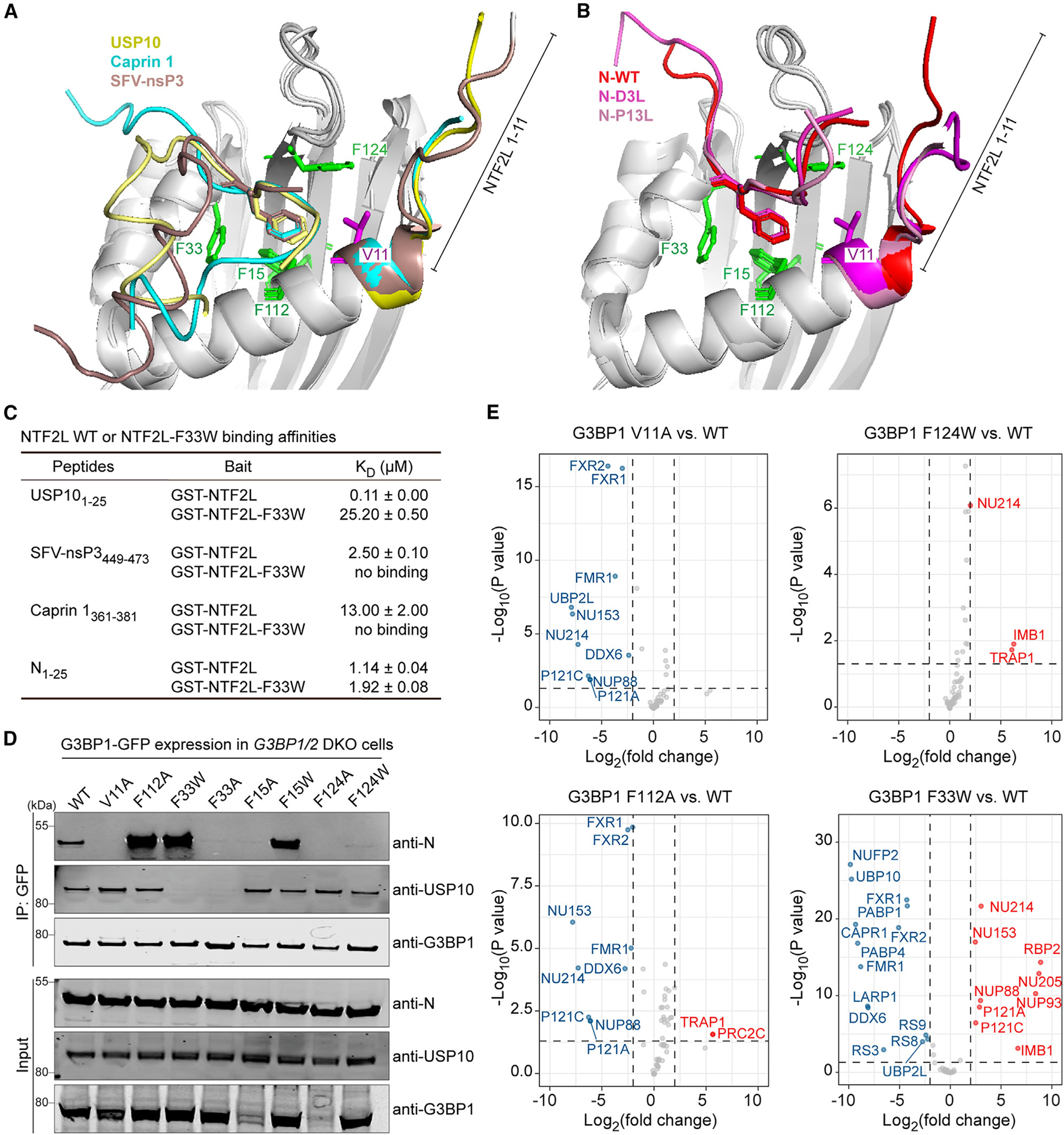
G3BP1-N interaction involves V11 and F124 of G3BP1 NTF2L (A and B) Alignment of crystal structures of G3BP1 NTF2L bound to USP10_2–25_ (PDB: 8TH6), caprin 1_361–381_ (PDB: 8TH7), and nsP3-SFV_449–473_ (PDB: 5FW5) (A) or bound to N-WT_1–25_ (PDB: 7SUO), N-D3L_1–25_ (PDB: 8TH1), and N-P13L_1–25_ (PDB: 8TH5) (B). The N-terminal tail (aas 1–11) of G3BP1 is labeled; side chains of G3BP1 F15, F33, F112, F124, and V11 are colored green (phenylalanines) or magenta (valine). (C) Results from the SPR assay, showing binding affinities of the indicated peptides with purified GST-tagged proteins. K_D_ values were derived from a global fit of all replicates ± SD. (D) HEK293T *G3BP1/2* DKO cells were co-transfected with N-FLAG and the indicated G3BP1-GFP constructs. Cell extracts were captured with magnetic beads conjugated with GFP antibody for IP, and bound proteins were analyzed by immunoblot. Representative images from two replicates are shown. (E) Comparison of the interactomes of G3BP1-WT with the indicated G3BP1 mutants in HEK293T *G3BP1/2* DKO cells. Dots indicate interactors that were significantly increased (red), decreased (blue), or unchanged (gray) in the interactome of the indicated G3BP1 mutant. An adjusted p < 0.05 and fold change of 2 were used as cutoffs. See also Table S2.

We next sought to identify the reciprocal residues of G3BP1 that are responsible for interaction with the N protein. Although all NTF2L-interacting peptides examined rely on a key phenylalanine to interact with G3BP1 (Figures 1 and S1), the aas surrounding this phenylalanine residue differ across the various NTF2L interactors, suggesting that the docking modes of these interactors on NTF2L are not identical. In caprin 1, USP10, and SFV-nsP3, we observed that aas around the key phenylalanine residue were in close proximity to a side of the NTF2L pocket made up of G3BP1 F33 and surrounding aas (Figure 3A). In contrast, the aas surrounding F17 on the N protein were in close contact with the opposing side of the NTF2L binding pocket, which has as its central components G3BP1 F124 and G3BP1 aas 1–11 (Figure 3B). These structural insights led us to hypothesize that it would be possible to engineer specific disruption of the G3BP1-N interaction without altering the interaction of G3BP1 with other host proteins.

We first examined the effect of mutating G3BP1 F33, the central aa within the NTF2L binding pocket used by caprin 1, USP10, and SFV-nsP3. Using SPR, we found that NTF2L-F33W abolished the binding of caprin 1_361–381_, USP10_1–25_, and nsP3_449–473_ peptides to NTF2L but had relatively little effect on the NTF2L-N_1–25_ interaction, as evidenced by only a minor decrease in binding affinity (K_D_ = 1.14 μM for NTF2L-WT, K_D_ = 1.92 μM for NTF2L-F33W) (Figure 3C). We next introduced single point mutations to impair the different surfaces of the NTF2L hydrophobic pocket, including V11, F15, F33, and F124, as well as F112, the phenylalanine very close to the binding surface. We found that G3BP1 V11A and F124A abolished the G3BP1-N interaction, and G3BP1 F124W, F15A, and F33A dramatically reduced the G3BP1-N interaction (Figure 3D). In contrast, G3BP1 F112A, F15W, and F33W showed increased G3BP1-N interaction (Figure 3D). These findings are consistent with a previous study in which the G3BP1 NTF2L domain with V11A, F15A, or F124A mutations lost the interaction with N peptide *in vitro*.^64^

To identify point mutations specifically targeting the N protein interaction, we excluded G3BP1 F33A/F33W because these mutations cause the loss of interaction with USP10 (Figure 3D) as well as G3BP1 F15A and F124A because these proteins were expressed at much lower levels, reflecting potential instability. We selected the remaining mutations (V11A, F112A, and F124W) for further unbiased analysis via mass spectrometry. For G3BP1 V11A, most of the 58 high-confidence interactors remained intact compared with G3BP1 WT. Notable exceptions included the loss of UBP2L and four Nups that harbor an FxFG motif (Nup153, Nup214, Nup88, Nup121) as well as a reduced interaction with FMR1 and FXR1/2 (Figure 3E; Table S2). G3BP1 F112A lost the interaction with these same four FxFG-containing Nups but not the other proteins affected by the V11A mutation. Overall, the interactome of G3BP1 F124W was similar to G3BP1 WT (Figure 3E). Ultimately, these studies identify three G3BP1 point mutations that alter the G3BP1-N interaction, albeit with different degrees of efficacy and specificity.

### N-F17A reduces SARS-CoV-2 replication in cultured cells

**Figure 4.**
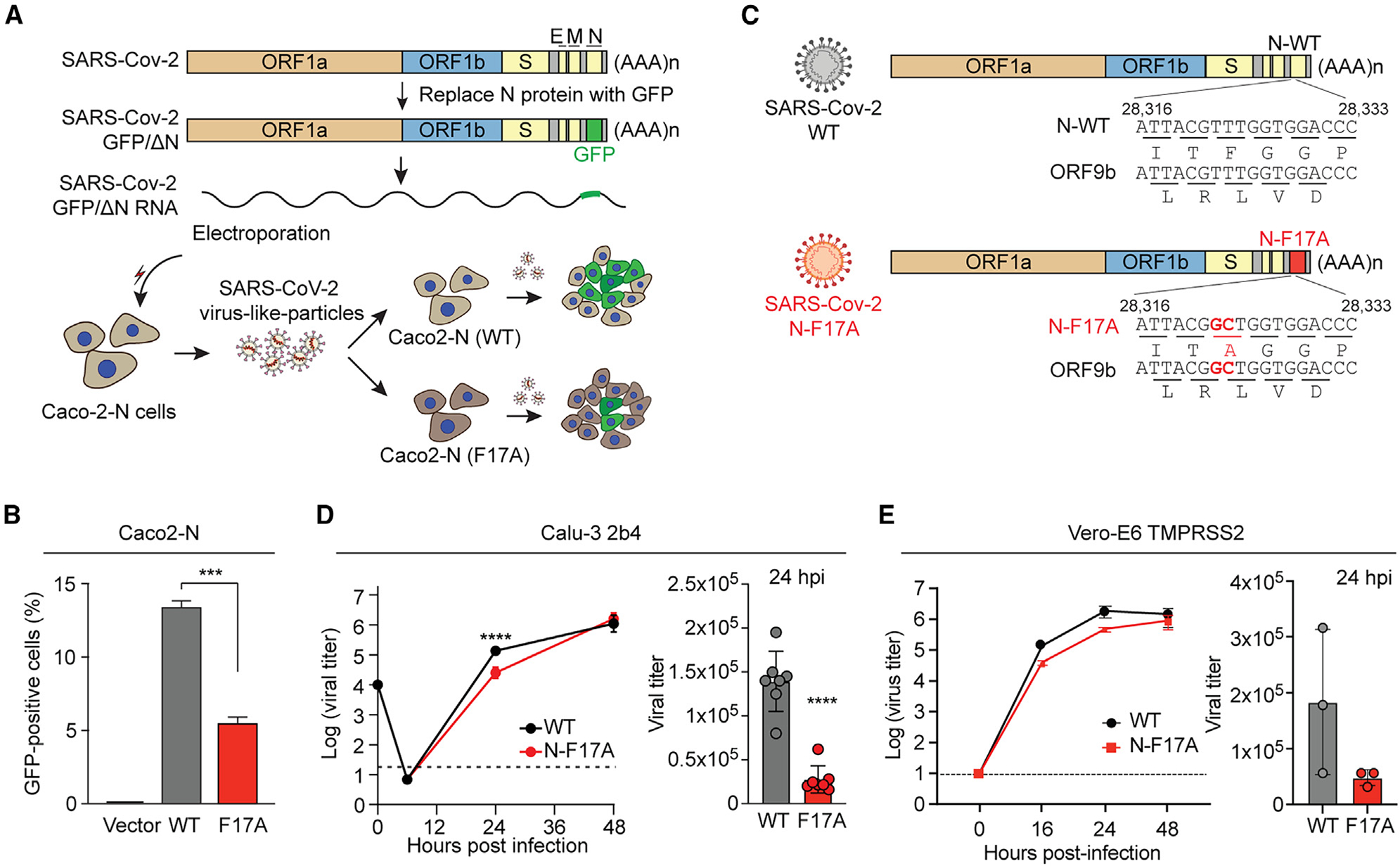
N-F17A reduces SARS-CoV-2 replication in cultured cells (A) Strategy for comparing SARS-CoV-2 GFP/ΔN trVLP reproduction in Caco2-N (WT) and Caco2-N (F17A) cells. SARS-CoV-2 GFP/ΔN RNA was transcribed *in vitro* and introduced into Caco2-N cells to produce infectious SARS-CoV-2 trVLPs. Caco2-N (WT) and Caco2-N (F17A) cells were infected by SARS-CoV-2 trVLPs (MOI 0.05, 36 h). The newly reproduced trVLPs in the supernatant of both cell lines were collected to infect fresh Caco2-N cells for 36 h. (B) Infected cells (as in A) were analyzed by flow cytometry to measure the percentage of GFP-positive cells. Error bars represent mean ± SD. ***p < 0.001 by one-way ANOVA with post hoc Tukey test. (C) Introduction of N-F17A into the SARS-CoV-2 genome. Open reading frames of both N and ORF9b are shown. (D and E) Calu-3 2b4 (D) and Vero E6-TMPRSS2 (E) cells were infected with WT or mutant SARS-CoV-2 (N-F17A) (MOI 0.01). Viral titers were determined at the indicated time points. Error bars represent mean ± SD. ****p < 0.0001 by two-tailed Student’s t test. See also Figure S3.

We next introduced the F17A mutation to a transcription- and replication-competent virus-like particle (trVLP)^72^ to evaluate the consequences of loss of the G3BP1-N interaction on SARS-CoV-2 infection (Figure 4A). In this N protein *trans*-complementation system,^72^ the *N* gene of the SARS-CoV-2 genome is replaced by a GFP reporter (SARS-CoV-2 GFP/ΔN), and the N protein is supplied in *trans* in Caco-2 cells (Caco2-N cells) to produce trVLPs suitable for low containment. To test the effect of the N-F17A mutation, we infected Caco2-N (WT) and N (F17A) cells with trVLPs (MOI 0.05, 36 h). The infectivity of newly reproduced trVLPs from Caco2-N (F17A) cells was significantly reduced compared with Caco2-N (WT) cells, as indicated by a more than 50% decrease in the percentage of GFP-positive cells among those inoculated with the cell culture supernatant (Figure 4B). These results indicate that N-F17A mutation attenuates viral replication in this system.

To corroborate this result using authentic SARS-CoV-2 virus, we introduced the N-F17A mutation into the Washington-1 (WA-1) strain using our SARS-CoV-2 reverse genetic system^73^ (Figure 4C). Introduction of the N-F17A mutation resulted in a replication-competent virus with no deficit in stock generation or change in focus morphology. After recovery of recombinant SARS-CoV-2, we measured the replication kinetics of the N-F17A mutant relative to WT SARS-CoV-2 in Calu-3 2b4 cells,^74^ a human respiratory cell line. Cells were infected with WT or the N-F17A mutant (MOI 0.01), and viral titer was determined over a 48-h period. The N-F17A mutant showed significantly reduced viral replication by more than 8-fold at 24 h post infection (hpi), indicating that the N-F17A mutation attenuates SARS-CoV-2 in human respiratory cells *in vitro* (Figure 4D). Similarly, in Vero E6-TMPRSS2 cells, African green monkey kidney cells constitutively expressing human transmembrane serine protease TMPRSS2,^75,76^ SARS-CoV-2 N-F17A virus replicated slightly less than the WT virus at 16 hpi, although the difference was not statistically significant (Figure 4E). In Vero E6 cells without TMPRSS2 expression, N-F17A mutant virus showed slightly enhanced replication compared with WT virus at 24 hpi (Figure S3), highlighting the importance of TMPRSS2 expression when evaluating SARS-CoV-2 infection *in vitro*.^77,78^ Thus, our findings demonstrate decreased replication of SARS-CoV-2 in infected cells due to the N-F17A mutation.

### N-F17A reduces SARS-CoV-2 replication and pathogenesis *in vivo*

**Figure 5.**
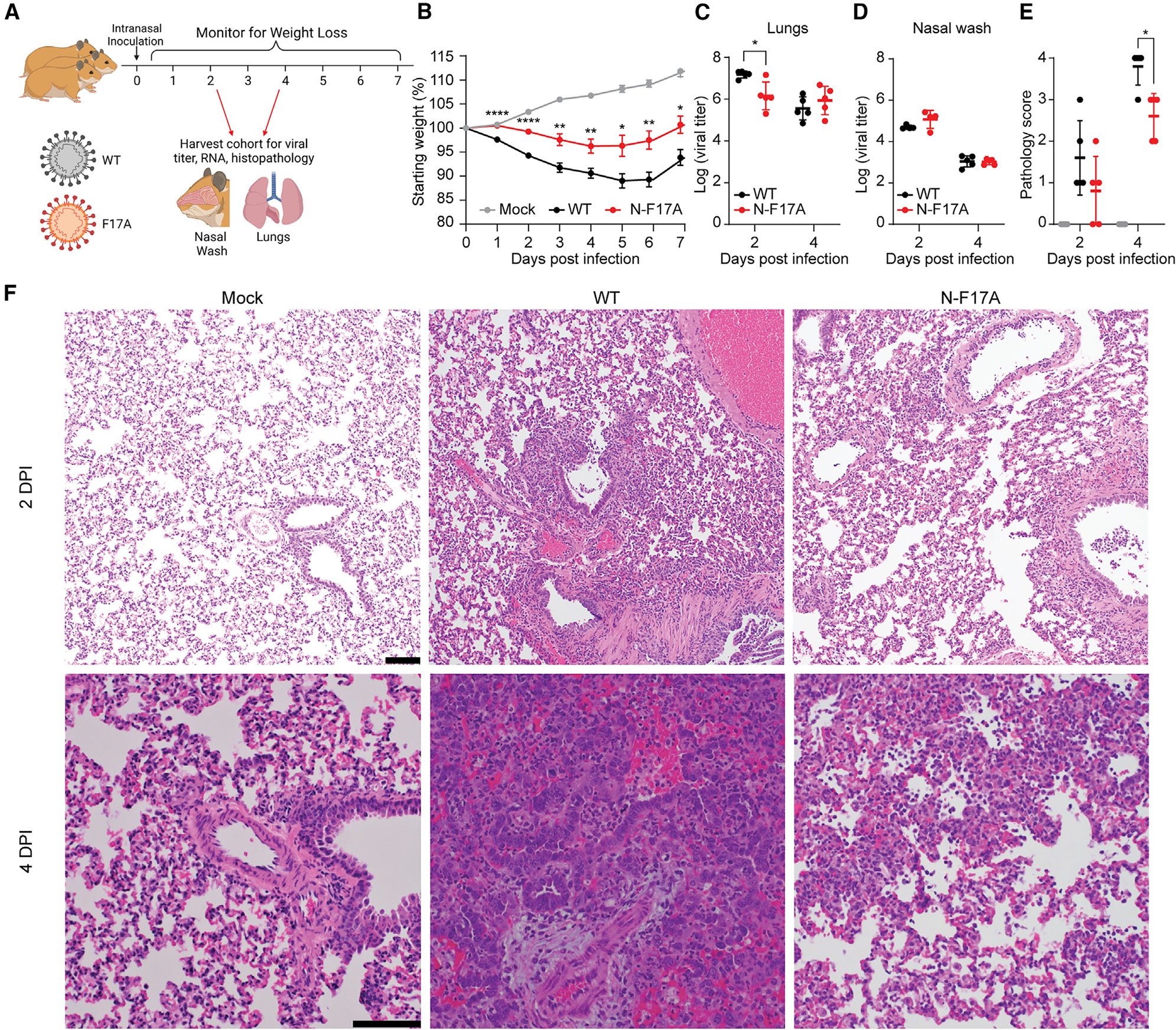
N-F17A reduces SARS-CoV-2 replication and pathogenesis *in vivo* (A) Three-to four-week-old male Syrian hamsters (n ≥ 5) were intranasally inoculated with PBS (mock) or PBS containing 10^5^ focus-forming units of WT or N-F17A mutant SARS-CoV-2. The illustration was generated using BioRender. (B) Weight change of animals post infection. Asterisks indicate statistical differences between WT and N-F17A. Data are shown as the mean ± SEM. (C–E) At 2 and 4 dpi, cohorts of 5 animals were nasally washed with PBS and sacrificed. Viral titers of lung tissue (C) and nasal wash (D) were analyzed by focus formation assay. Symbols represent individual subjects, midlines the mean, and error bars ±SD. Lung tissue pathology (E) was analyzed by H&E and scored by a blinded pathologist. (F) Representative H&E staining of lung tissues at 2 and 4 dpi. Scale bar, 100 μm. *p < 0.05, **p < 0.01, ****p < 0.0001 by two-tailed Student’s t test.

We next evaluated the effect of the N-F17A mutation *in vivo* using Syrian hamsters, which have proven useful in modeling SARS-CoV-2 pathogenesis due to their faithful recapitulation of lung pathology observed in human disease.^79–81^ As described previously,^82^ 3- to 4-week-old male Syrian hamsters were intranasally infected with either PBS alone (mock), WT, or N-F17A SARS-CoV-2 and monitored for disease through 7 days post infection (dpi) (Figure 5A). At 2 and 4 dpi, animals underwent nasal washing followed by euthanasia; lungs were harvested to assay viral replication and histopathologic changes. Notably, N-F17A-infected animals had significantly reduced weight loss relative to WT-infected animals throughout the experimental period, demonstrating reduced pathogenesis (Figure 5B). Though the N-F17A mutant had no effect on viral titer in the nasal washes, N-F17A-infected animals had significantly lower viral titers in the lungs at 2 dpi compared with WT-infected animals (Figures 5C and 5D). In addition, histopathological analysis of lung tissue revealed a significant reduction in overall lesions in N-F17A-infected animals compared with WT-infected animals at 4 dpi (Figure 5E). Compared with mock infection, WT-infected animals exhibited widespread interstitial pneumonia, mononuclear cell infiltration of alveolar septa, and numerous enlarged cytopathic alveolar pneumocytes with multinucleation and prominent nucleoli (Figure 5F). In contrast, N-F17A-infected animals exhibited patchy areas of interstitial pneumonia with few cytopathic alveolar pneumocytes (Figure 5F). These data demonstrate that the N-F17A mutation attenuates SARS-CoV-2 replication and pathogenesis *in vivo*.

### N protein rewires the G3BP1 network to free viral gRNA from being diverted into condensates

**Figure 6.**
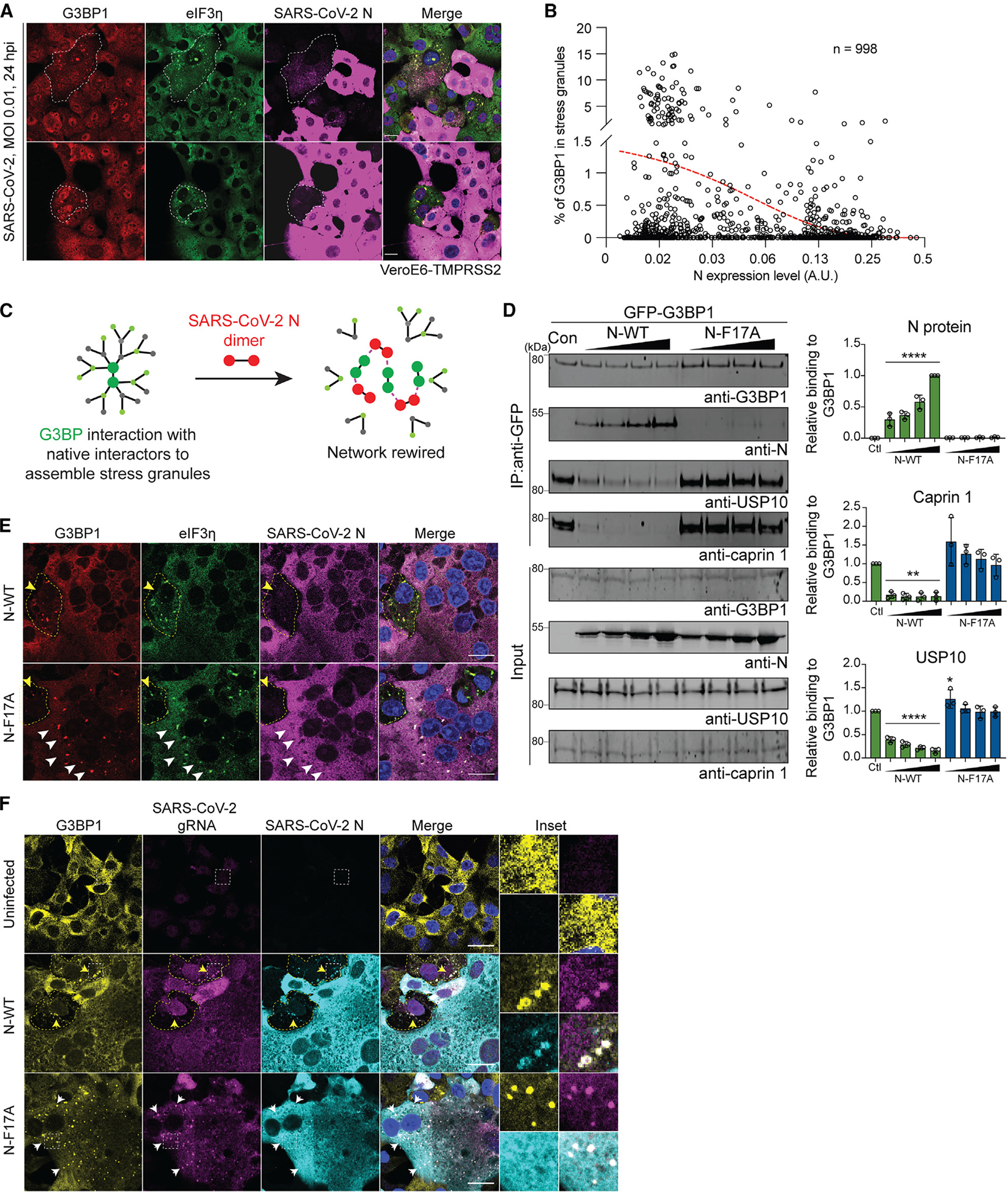
N protein rewires the G3BP1 network to free viral gRNA from being diverted into condensates (A and B) Vero E6-TMPRSS2 cells were infected with SARS-CoV-2 (MOI 0.01, 24 h) and fixed. Images of 998 infected cells were analyzed quantitatively. N protein expression levels were measured by antibody staining intensity, and SG formation was measured by the enrichment ratio of G3BP1 inside eIF3η-positive puncta (B). A red trendline indicates the inverse correlation between N expression level with G3BP1 enrichment inside SGs. Scale bars, 20 μm. (C) Illustration of how N protein may rewire the G3BP1-centered protein interaction network. (D) HEK293T *G3BP1/2* DKO cells were transfected with G3BP1-GFP and lysed after 48 h. Increasing amounts of purified N-WT and N-F17A proteins were added to the cell extracts, which were further captured with magnetic beads conjugated with GFP antibody for IP. Bound proteins were analyzed by immunoblot. Representative images and quantification of three replicates are shown. Error bars represent mean ± SD. **p < 0.01, ****p < 0.0001 by one-way ANOVA. (E) Vero E6-TMPRSS2 cells were infected with WT or N-F17A mutant SARS-CoV-2 (MOI 0.01). Cells were fixed for staining with the indicated antibodies at 24 h post infection (hpi). Yellow and white arrowheads indicate infected cells at early and late stages of infection, respectively, distinguished by the expression levels of viral N proteins. Scale bars, 20 μm. (F) Vero E6-TMPRSS2 cells were infected as in (E). At 24 hpi, cells were stained for viral gRNA, G3BP1, and N. For cells infected with WT SARS-CoV-2, manual counting was used to quantify the number of cells in which viral gRNA was concentrated within G3BP1 condensates. Multiple images from different fields were stitched together to create a single composite image, providing a broader view. Yellow and white arrows indicate infected cells at early and late stages of infection, respectively, distinguished by the expression levels of viral N proteins. Scale bars, 20 μm. See also Figure S4.

SARS-CoV-2 N protein expression dramatically increases as infection progresses, becoming the most abundant viral protein in infected cells within 24 hpi.^7–13^ Indeed, a quantitative proteomics analysis in SARS-CoV-2-infected (MOI 0.3) Vero E6 cells revealed that the abundance of N protein is ~12-fold higher than that of G3BP1 and 30-fold higher than that of G3BP2 at 24 hpi, as estimated by the number of peptide spectrum matches.^83^ To investigate the effects of different expression levels of N protein, we infected Vero E6-TMPRSS2 cells with SARS-CoV-2 and assessed their relative expression of N and G3BP1 inside G3BP1 condensates. We observed an inverse relationship between N protein expression levels and G3BP1 condensate formation, where cells with low levels of N protein tended to form G3BP1 condensates, whereas those with high levels of N protein showed a reduced propensity for G3BP1 condensate formation (Figure 6A). We confirmed this inverse correlation through quantitative imaging of 998 infected cells, in which N protein expression levels were measured by antibody staining intensity, and G3BP1 condensates were measured using the enrichment ratio of G3BP1 inside eIF3η-positive puncta (Figure 6B).

To determine the extent to which N protein expression, among many other changes in infected cells, and its interaction with G3BP1, was specifically suppressing G3BP1-dependent condensate formation, we expressed GFP-tagged N protein in G3BP1-tdTomato-KI U2OS cells and induced SG formation using sodium arsenite. We observed impaired SG formation in cells with high expression of N-WT-GFP but not in cells with high expression of N-F17A-GFP (Figures S4A and S4B), consistent with a direct role of N protein in suppressing G3BP1-dependent condensate formation. In cells with moderate or low expression of N-GFP, we observed the recruitment of N-WT and N-F17A into SGs but no significant impairment in SG formation (Figures S4A and S4B). These results suggest that high levels of N protein suppress G3BP1-dependent condensate formation, and this requires G3BP1-N interaction. Thus, we hypothesized that increasing N protein expression during SARS-CoV-2 infection competes with native G3BP1-interacting proteins for binding to G3BP1. This competitive binding by the N protein “rewires” the G3BP1-centered network and thereby inhibits SG formation (Figure 6C). Indeed, coIPs from cells demonstrated that G3BP1 increasingly lost interaction with USP10 and caprin 1 as increasing amounts of N-WT protein were added, but this effect was not observed with the addition of N-F17A (Figure 6D).

We next sought to define how SARS-CoV-2 might benefit from elimination of G3BP1 condensates. In SGs induced by environmental stress, longer mRNAs, which provide more binding valency for G3BP1/2 and other RNA-binding proteins,^20^ are preferentially enriched inside SGs compared with shorter mRNAs.^84^ In addition, RNAs that are particularly abundant are more likely to initiate G3BP1-RNA condensation in cells.^20^ Applying this concept to G3BP1-dependent condensates during SARS-CoV-2 infection, we hypothesized that long and abundant viral RNAs, particularly viral gRNAs, which are long (~30 kb) and abundant in infected cells,^12,85^ would be diverted to G3BP1 condensates.

To test whether viral gRNA is sequestered in G3BP1 condensates, we infected VeroE6-TMSSPR2 cells with WT or N-F17A SARS-CoV-2. As predicted, infection with SARS-CoV-2 F17A led to persistent G3BP1 condensates even during late stages of infection, when the N protein was highly expressed and cells began to exhibit syncytium morphology (Figure 6E), suggesting an inability to disassemble SGs. We then co-stained infected cells for G3BP1 and viral gRNA, labeling the latter using a combination of probes targeting different conserved regions of OR-F1ab.^86^ Following infection with WT SARS-CoV-2, G3BP1 condensates showed enrichment of viral gRNA in approximately half of the cells (Figure 6F). Following infection with SARS-CoV-2 F17A, viral gRNA sequestration was much more pronounced within the persistent G3BP1 condensates (Figure 6F).

The diversion of viral gRNAs, and possibly other long viral RNAs, into G3BP1 condensates, an environment where viral RNA remains inaccessible to translational machinery, may lead to a reduction in viral replication. We suggest that SARS-CoV2 exploits the G3BP1-N interaction to mitigate G3BP1-dependent condensation of viral gRNAs. In the case of SARS-CoV-2 N-F17A, the G3BP1-N interaction is disrupted, causing the virus to lose its ability to disassemble G3BP1 condensates. Consequently, the viral gRNAs remain sequestered within these persistent G3BP1 condensates, ultimately resulting in a decrease in viral replication.

## DISCUSSION

G3BP1/2 acts as surveillance machinery in the host cell cytoplasm, where increasing concentrations of uncoated mRNAs trigger the condensation of RNAs with proteins into SGs.^20^ When this process is triggered, G3BP1/2 serves as a hub for proteins that create a network of interactions to promote the formation of a phase-separated RNP granule. Most of these interactions occur in a mutually exclusive manner via a common binding pocket in G3BP1/2 NTF2L. We found that the N protein exploits the critical nature of this interaction surface by filling the NTF2L binding pocket with residue F17, thereby impairing the ability of G3BP1 to form the SG interaction network.

Our study further shows that SARS-CoV-2 F17A has reduced replication, consistent with G3BP1 knockdown studies showing modest increases in WT SARS-CoV-2 viral RNA accumulation.^41^ Our *in vivo* data demonstrate a major shift in disease, with F17A-infected hamsters showing reduced weight loss, attenuated viral replication in the lungs, and less overall immune infiltration. While mice with conditional *G3BP1* KO in type II alveolar cells had augmented SARS-CoV-2 RNA early during infection, no changes in disease were reported.^41^ The limited impact of G3BP1 KO on infection in these mice suggests that, as N protein accumulates, it increasingly controls G3BP1 activity. In contrast, loss of G3BP1 antagonism via the N-F17A mutation results in significant attenuation of pathogenesis and is among the most robust demonstrations of SG antiviral activity *in vivo*. Together, these results highlight the importance of the G3BP1-N interaction in controlling SG formation during SARS-CoV-2 pathogenesis.

While these studies demonstrate the importance of condensation for SARS-CoV-2, the underlying mechanisms are complex. We observed the formation of transient G3BP1 condensates in early stages of infection, a common observation in RNA virus infection.^87^ These transient condensates, which also showed positivity for eIF3η, an SG marker, were rapidly disassembled as N-WT protein levels increased. In contrast, SARS-CoV-2 mutant N-F17A increased the formation of persistent G3BP1 condensates that were enriched with viral RNA. This suggests that viral replication is attenuated by sequestration of viral RNA into condensates. Another explanation is that G3BP1 condensates are involved in initiating innate immune responses; however, prior reports have been mixed, finding that SGs can amplify,^88,89^ regulate,^90^ or have no role in innate immune signaling.^91^

A third possibility is that the N protein co-opts host G3BP1 protein for pro-viral activity independent of its role in the dissolution of SGs. For example, one recent study demonstrated that overexpression of a peptide derived from SFV-nsP3, which binds G3BP1 NTF2L and suppresses SG formation,^92^ suppressed SARS-CoV-2 replication in Vero E6 cells.^46^ These findings are consistent with our observation that G3BP1 facilitates phase separation of N protein (Figure S5). Specifically, we found that, although N protein did not undergo phase separation alone at physiological salt concentrations, the addition of purified G3BP1 induced co-condensation with N-WT, but not N-F17A, and lowered the concentration threshold for co-condensation of N protein and RNA. In this context, the G3BP1-N interaction may aid engagement with viral RNAs and have pro-viral effects, including increased viral RNA replication/translation and packaging of viral gRNA. These potential antiviral and pro-viral activities are not mutually exclusive but, rather, highlight the importance of the G3BP1-N interaction throughout various stages of SARS-CoV-2 infection. Thus, we propose that the potential roles of G3BP1 as both an antiviral and pro-viral factor are not inherently conflicting but, rather, contingent on its interactions with specific protein partners. When G3BP1 predominantly interacts with its native SG-associated partners during the early stages of infection, G3BP1 serves an antiviral role by triggering SG formation. Conversely, when primarily interacting with the viral N protein, which is more prevalent during the later stages of infection, G3BP1 assumes a pro-viral role by assisting the N protein in viral replication processes (Figure S6).

Our study defines the key residues that govern the G3BP1-N interaction, providing insights into SARS-CoV-2 variant attenuation and avenues for potential treatment. While the G3BP1-NTF2L domain serves as an interaction hub for both the SARS-CoV-2 N protein and host proteins, we found that the interface between NTF2L and N is distinct from that utilized by other host proteins. Mutation of F124 in G3BP1 selectively impaired interaction with the N protein while maintaining the G3BP1 host interactome. These findings suggest that targeting this unique interface on the G3BP1-NTF2L domain might offer an antiviral approach with limited toxicity. In the N protein, while the N-terminal domain is highly disordered, F17 resides between two regions that give rise to transient helices and may offer a target site for drug design.^1^ Notably, we found that the Omicron variant mutation P13L reduced the G3BP1-N interaction. This suggests that targeting sites adjacent to F17 in the N protein may alter G3BP1 binding and contribute to reduced virulence observed in Omicron variants.^93–96^ Further studies are needed to evaluate targeting G3BP1-N interaction as a SARS-CoV-2 antiviral intervention.

### Limitations of the study

We have demonstrated that the N-F17A mutation causes highly selective loss of interaction with G3BP1/2 without otherwise altering key features of the N protein, including its interactome, its RNA-binding ability, and its phase separation behavior with purified protein *in vitro*. Nonetheless, given the complex and dynamic interactions between host and virus that occur during infection *in vivo*, it remains formally possible that the F17A mutation may have other effects on the virus independent of its altered interaction with G3BP1/2.

## STAR★METHODS

### RESOURCE AVAILABILITY

#### Lead contact

Further information and requests for resources and reagents should be directed to and will be fulfilled by the lead contact, J. Paul Taylor (jpaul.taylor@stjude.org).

#### Materials availability

Unique materials generated in this study are available upon request from the lead contact, J. Paul Taylor (jpaul.taylor@stjude.org).

#### Data and code availability

- Original data generated in this study are available upon request from the lead contact, J. Paul Taylor (jpaul.taylor@stjude.org). The reported structures were deposited in the Protein Data Bank. Accession numbers are listed in the Key resources table. Access to any additional information in this study is available upon request from the lead contact, J. Paul Taylor (jpaul. taylor@stjude.org).

### EXPERIMENTAL MODEL AND STUDY PARTICIPANT DETAILS

#### Hamster infections

Three-to four-week-old male Syrian hamsters (HsdHan:AURA strain) were purchased from Envigo. Animals were intranasally inoculated with 100 mL PBS (mock) or PBS containing 10^5^ focus forming units (ffu) of WT or F17A mutant SARS-CoV-2. Animals were monitored for weight loss and signs of disease for 7 days. On 2 and 4 days post infection (dpi), cohorts of 5 animals were anesthetized with isoflurane and nasal washed with PBS and then sacrificed. Lung tissue was then taken to analyze viral titer by focus forming assay and pathology by hemoxylin and eosin (H&E) staining. H&E staining was performed by the University of Texas Medical Branch Histology Laboratory and then analyzed and scored by a blinded pathologist.

All animal studies were carried out in accordance with a protocol approved by the University of Texas Medical Branch Institutional Animal Care and Use Committee and complied with United States Department of Agriculture guidelines in a laboratory accredited by the Association for Assessment and Accreditation of Laboratory Animal Care. Procedures involving infectious SARS-CoV-2 were performed in the Galveston National Laboratory animal biosafety level 3 (ABSL3) facility.

### METHOD DETAILS

#### Cell culture and transfection

HEK293T and G3BP1-tdTomato-KI U2OS^20^ cells were cultured in Dulbecco’s modified Eagle’s medium (HyClone), supplemented with 10% fetal bovine serum (HyClone). Vero E6 and Vero E6-TMPRSS2 (XenoTech; JCRB1819) cells were cultured with DMEM (Gibco; 11965–092), supplemented with 10% fetal bovine serum and 1% antibiotic/antimitotic (Gibco; 5240062). Calu-3 2b4 cells were grown in DMEM with 10% defined FBS, 1% antibiotic/antimitotic, and 1 mg/mL sodium pyruvate. Transient transfections were performed using Lipofectamine 2000 (Thermo Fisher; 11668019), following the manufacturer’s instructions.

#### Constructs

Full-length cDNA of the N protein was synthesized using the SARS-CoV-2 genomic reference sequences, but with alternative codons (Integrated DNA Technologies). N-FLAG was inserted into the backbone of pEGFP-C3 plasmid (with GFP removed). AcGFP-N was inserted into the backbone of pAcGFP1-C1 plasmid. His-SUMO-N was inserted into the backbone of pETite-HisSUMO plasmid (Lucigen) using NEBuilder HiFi DNA Assembly Master Mix kit (NEB; E2621). Similarly, human G3BP1 cDNA was inserted into the pGEX-2T plasmid. G3BP1-EGFP, mCherry-nsP3–25mer (449–472) were inserted into the backbone of pEGFP-C3 plasmid (with GFP removed); EGFP-N-25mer (1–25), EGFP-caprin1–21mer (361–381), EGFP-USP10–25mer (1–25), EGFP-nsP3–25mer (449–473) were inserted into the backbone of pEGFP-C3 plasmids. Truncations of N-FLAG (48–419, 175–419, 210–419, 1–361, 1–267, 1–209), GST-G3BP1 (GST-NTF2L, GST-deltaNTF2L), and AcGFP-N (1–47, 1–35, 1–25) were created using the NEBuilder HiFi DNA Assembly Master Mix kit. Point mutations of N-FLAG (F17A and F17W), AcGFP-N (F17A), GFP-tagged peptides (EGFP-N-25mer-F17A, EGFP-caprin1–21mer-F372A, EGFP-USP10–25mer-F10A and EGFP-nsP3–25mer-F451/468A), GST-NTF2L (F33W), G3BP1-GFP (V11A, F112A, F33A, F33W, F15A, F15W, F124A, F124W) were created using the Q5 Site-Directed Mutagenesis kit (NEB; E0554S).

#### Generation of HEK293T G3BP1/2 DKO cell line

To generate HEK293T *G3BP1/2* DKO cells, gRNAs targeting G3BP1/2 were designed through http://crispr.mit.edu/. The synthesized DNA oligos for gRNAs were then ligated into BbsI-digested px459 vectors (Addgene; 62988).^100^ Parental HEK293T cells were transiently transfected with gRNA vector and cultured for 2 days. After that, 2 μg/mL puromycin was added to the culture medium for a duration of 3 days to select for cells that successfully integrated the gRNA vector. To obtain KO clones, single-cell dilution was performed in 96-well plates. The successful KO was confirmed by antibody staining and subsequent immunoblot analysis.

#### Immunoblotting

Cells were washed twice with PBS and lysed with RIPA buffer (25 mM Tris-HCl, pH 7.6, 150 mM NaCl, 1% NP-40, 1% sodium deoxycholate, 0.1% SDS; Thermo Fisher; 89901), supplemented with a proteinase inhibitor cocktail (Roche; 1183617001). After 15 min of centrifugation at 4°C and 20,000 × g, the supernatant was collected and boiled with NuPAGE LDS sample buffer (Thermo Fisher; NP0007) at 90°C for 5 min. The samples were separated in 4–12% NuPAGE Bis-Tris gels (Thermo Fisher; NP0336BOX or NP0321BOX) and transferred to PVDF membranes (Thermo Fisher; IB24001) using an iBlot 2 transfer device (Thermo Fisher). Membranes were blocked with Odyssey blocking buffer (LI-COR Biosciences; 927–50000) and then incubated with primary antibodies at 4°C overnight. Primary antibodies used in this study were anti-G3BP1 (mouse; BD Biosciences; 611127), anti-G3BP1 (rabbit; Proteintech; 13057–2-AP), anti-G3BP2 (rabbit; Thermo Fisher; PA5–53776), anti-N (mouse; Sino Biological; 40143-MM05), anti-N (rabbit; Sino Biological; 40143-R019), anti-USP10 (Rabbit; Proteintech; 19374–1-AP), anti-caprin 1 (Rabbit; Proteintech; 15112–1-AP), anti-GST (rabbit; BioVision; 3997–30T), anti-FLAG M2 antibody (mouse; Sigma-Aldrich; F1804), anti-mCherry (Rabbit; BioVision; 5993), anti-Hisx6 tag (mouse; abcam; ab18184), anti-GFP (Rabbit, Cell Signaling Technology; 2555). Membranes were washed three times with TBS-T (0.05% Tween) and further incubated with IRDye 680RD/800CW-labeled secondary antibodies (LI-COR Biosciences; 926–68073 or 926–32212) at a dilution of 1:10,000. Membranes were visualized with an Odyssey Fc imaging system (LI-COR Biosciences).

#### Immunoprecipitation

Cells were washed twice with PBS and lysed with IP lysis buffer (25 mM Tris-HCl, pH 7.4, 150 mM NaCl, 1% NP-40, 10% glycerol) supplemented with proteinase inhibitor cocktail (Roche; 1183617001). Lysates were centrifuged at 4°C for 15 min at 20,000 × g. Supernatants were incubated with anti-FLAG M2 magnetic Beads (Millipore; M8823) or GFP-Trap magnetic beads (ChromoTek; gtma10) at 4°C overnight. Beads were washed three times with lysis buffer, with the second wash supplemented with 0.1 mg/mL RNaseA (Thermo Fisher; EN0531) to remove RNA and hence the RNA-mediated protein-protein interactions. The beads were boiled in 1× NuPAGE LDS sample buffer at 70°C for 5 min and analyzed by immunoblotting.

#### Protein interactome by LC-MS/MS

Proteins interacting with GFP-N-WT, GFP-N-F17A, G3BP1-GFP WT, and G3BP1-GFP mutants were immunoprecipitated as described above. Resulting samples were isolated from gels and digested with trypsin overnight. Samples were loaded on a nanoscale capillary reverse-phase C18 column by an HPLC system (Thermo Ultimate 3000) and eluted by a gradient (~90 min). Eluted peptides were ionized by electrospray ionization and detected by an inline mass spectrometer (Thermo Orbitrap Fusion). Database searches were performed using SEQUEST search engine using an in-house SPIDERS software package. Tandem mass spectrometry spectra were filtered by mass accuracy and matching scores to reduce the protein false discovery rate to ~1%. The total number of spectral counts for each protein identified was reported by sample. The p value of spectral count changes between different groups was derived by G-test.^101^ Proteins were accounted as confident N protein interactors if they were repeatedly found in all four independent assays. A composite protein-protein interaction (PPI) network was built by combining STRING.^102^ For the comparison of two interactomes, proteins with spectral count >2, at least 2-fold change, and the p value less than 0.05 were considered as significant changes.

#### RNA cross-linking immunoprecipitation

The binding of N-WT and N-F17A with cellular RNA was tested as previously described.^20^ Briefly, HEK293T cells transfected with pAcGFP-N-WT or pAcGFP N-F17A were washed with PBS and exposed to UV (254 nm, 400 mJ/cm^2^ followed by 200 mJ/cm^2^). Cells were then harvested in lysis buffer containing 20 mM Tris HCl, pH 7.5, 137 mM NaCl, 1% Triton X-100, 2 mM EDTA, 1x protease inhibitor and incubated for 10 min on ice. Lysates were then successively treated with 8 U/mL DNase I (NEB; M0303S) for 5 min at 37°C and 4 U/mL RNase I for 3 min at 37°C. The reaction was stopped by the addition of 0.4 U/μL RNaseIn (Promega; N2615). Lysates were then centrifuged at 21,000 × g for 10 min at 4C. The supernatant fraction was incubated with 10 μL GFP-Trap magnetic beads at 4°C overnight. The beads were washed twice with lysis buffer, twice with 1 M NaCl, and twice with lysis buffer again. Beads were further suspended in 100 μL 10 mM Tris-HCl, pH 7.5, and treated with 2 units of calf intestinal alkaline phosphatase (Promega; M182A) at 37°C for 10 min at 1000 rpm in a Thermomixer. Beads were washed with lysis buffer twice and RNA labeling was performed with an RNA 3ʹ End Biotinylation Kit (Pierce; 20160). After washing with lysis buffer twice, beads were boiled in 1x LDS sample buffer (Life Technologies; NP007) with 100 mM DTT. Protein-RNA complexes were separated by 4–12% NuPAGE Bis-Tris gels, transferred to nitrocellulose membranes, and blotted with IRDye 680LT streptavidin (LI-COR; 926–68031).

#### Protein purification

GST-G3BP1 full-length and mutants, His-SUMO-N-WT and His-SUMO-N-F17A were expressed and purified from *E. coli* Rosetta 2(DE3) cells (Millipore; 71400–4). *E. coli* transformed with indicated expression plasmids were grown to OD_600_ of 0.8 and induced with 1 mM IPTG at 16°C overnight. Pelleted cells were resuspended in lysis buffer (300 mM NaCl, 50 mM HEPES 7.5, 1 mM DTT, protease inhibitor). After sonication, lysates were pelleted at 30,000 × g at 4°C for 30 min. For GST-tagged G3BP1 proteins, supernatants were applied to packed Glutathione Sepharose 4B resin (Cytiva; 17–0756-01) and were eluted with 10 mM glutathione (Sigma-Aldrich; G4251) in lysis buffer. For His-SUMO-tagged proteins, the lysis buffer was supplemented with 60 mM imidazole. Cell lysate supernatants were applied to packed Ni-NTA beads (QIAGEN; 30210). After washing with 20 column volume of lysis buffer, the proteins were eluted with 300 mM imidazole in lysis buffer. All eluted proteins were further treated with 0.1 mg/mL RNaseA to remove RNA.^103^ The fractions were analyzed by SDS-PAGE, pooled, and concentrated. For proteins that needed tags removed, TEV protease^104^ or SUMO Express proteinase (Lucigen, 30801–2) were respectively incubated with GST-TEV-tagged G3BP1 proteins and His-SUMO-tagged N proteins at 4°C overnight. The proteins were then purified by Superdex 200 16/200 column (GE) equilibrated in SEC buffer (300 mM NaCl, 50 mM HEPES 7.5, 1 mM DTT). The fractions were analyzed by SDS-PAGE, pooled, concentrated, filtered, flash frozen in liquid nitrogen, and stored at −80°C.

#### Peptide synthesis

All peptides were synthesized by the Hartwell Center for Bioinformatics and Biotechnology at St. Jude Children’s Research Hospital, Molecular Synthesis Resource, using standard solid-phase peptide synthesis chemistry. The peptides were reconstituted from lyophilized form into DMSO for subsequent experiments.

#### Pulldown

GST-tagged proteins (100 nM), including GST-G3BP1-full length, GST-NTF2L, GST-NTF2L-F33W, and GST-DNTF2L, were incubated with His-SUMO tagged proteins (100 nM), including His-SUMO-N-WT or His-SUMO-N-F17A, for 1 h at room temperature in binding buffer (50 mM HEPES, pH 7.5, 150 mM NaCl, 1% Triton X-100, 1 mM DTT). Complexes were pulled down using Glutathione Sepharose 4B resin (Cytiva; 17–0756-01) or Ni-NTA resin (QIAGEN; 30210) for 2 h at 4°C, washed 3 times with binding buffer, with the second wash supplemented with 0.1 mg/mL RNaseA to remove RNA and hence RNA-mediated protein-protein interactions. Beads were boiled in 13 NuPAGE LDS sample buffer at 70°C for 5 min and analyzed by Coomassie blue staining or immunoblotting with anti-GST and anti-6xHis antibodies.

#### Crystallography studies and structure analysis

The NTF2L domain of G3BP1 (10 mg/mL) co-crystallized with Caprin1 22mer (3X molar ratio) at 18°C in 1.8M tri-ammonium citrate pH 7.0. NTF2L at 7.5 mg/mL or 10 mg/mL co-crystallized at 4°C in presence of 7.5X excess of the SARS-CoV-2 N 25mer peptide harboring a D3L or P13L mutant, respectively. D3L co-crystals appeared in 0.2 M sodium thiocyanate and 20% PEG 3350, while P13L crystals appeared in 0.2 M lithium sulfate, 0.1 M Tris pH 8.5, 25% PEG3350. Finally, the NTF2L domain (10 mg/mL) co-crystallized with 3X USP10 24mer in 0.1 M HEPES pH 7.5, 25% PEG3350 at 4C. All drops were set up as a 1:1 mixture of protein to reservoir solution. Crystals were cryoprotected with 10–15% glycerol and flash cooled. The caprin 1, N- D3L, N-P13L, and USP10 datasets were collected at 1.18057Å (CLS), 1.03319 Å (PETRAIII), 1 Å (Spring-8) and 0.97915 Å (SSRF) respectively.

Data processing and scaling were performed with DIALS.^105^ The diffraction data was phased by molecular replacement using PDB 5FW5,^60^ as the search model. The initial model was built by Phaser MR^97^ and completed with Coot.^98^ Refmac^99^ was used to improve density between rounds of manual building. Data collection and refinement statistics are shown in Table S1. Coordinates of structures from this study have been deposited in the Protein DataBank with the accession codes 8TH7 (NTF2L/caprin 1_360–381_), 8TH1 (NTF2L/N-D3L_1–25_), 8TH5 (NTF2L/N-P13L_1–25_), 8TH6 (NTF2L/USP10_2–25_). Crystallographic data and refinement statistics are provided in Table S3. Structure analysis and images of binding pockets were made in PyMol. Crystal structures of NTF2L bound to peptides 5FW5 (SFV-nsP3), 8TH7 (caprin 1), 8TH1 (N-D3L), 8TH5 (N-P13L), 8TH6 (USP10), and 4FCM (Nup FxFG) were aligned to the WT-Apo structure (4FCJ) using the “super” method and only aligning backbone atoms.

#### Surface plasmon resonance

Binding studies were performed at 25°C using a Biacore T200 optical biosensor (Cytiva). Anti-GST antibodies (Cytiva) were covalently attached to a carboxymethyl dextran-coated gold surface (CM-4 Chip; Cytiva). GST-tagged NTF2L constructs were captured on the flow cell surfaces, and GST was captured on the reference surface to account for any non-specific binding to the GST tag. The peptide analytes were prepared in 20 mM HEPES (pH 7.5), 150 mM NaCl, 5% glycerol, 0.01% Triton X-100, 5% DMSO as 3-fold dilution series and injected in triplicate at flow rate 75 μL/min. Data were processed, double-referenced, solvent corrected, and analyzed using the software package Scrubber 2 (Version 2.0c, BioLogic Software). Equilibrium dissociation constants (K_D_) were determined by fitting the data to a 1:1 (Langmuir) binding model.

#### Liquid-liquid phase separation

*In vitro* LLPS experiments were performed at room temperature. Indicated concentrations of protein(s), RNA, or Ficoll 400 were mixed together in low binding tubes (COSTAR; 3206) and transferred to a sandwiched chamber created by cover glass and a glass slide with a double-sided spacer (Sigma-Aldrich; GBL654002). Samples were observed under a DIC microscope using a Leica DMi8 microscope with a 20x objective. All imaged were captured within 5 min after LLPS induction. RNA used in LLPS assays was isolated from HEK293T cells using TRIzol (Thermo Fisher; 15596026) and the concentration of RNA was measured by Nanodrop (Thermo Fisher).

#### SARS-CoV-2 N protein amino acid variation analysis

SARS-CoV2 genome sequences (n = 8,297,154) deposited in GISIAD^70^ were accessed in March 2022. N protein residue usage analyses were based on multiple sequence alignments. Residues used in the N protein of each genome were enumerated and fraction of residue usage was derived via customized scripts.

#### SARS-CoV-2 GFP/ΔN trans-complementation cell culture system

The reproduction of transcription and replication-competent virus-like-particles in Caco2-N (WT) and Caco2-N (F17A) cells was tested as previously described.^72^ Briefly, five DNA fragments of SARS-CoV-2 GFP/ΔN, SARS-CoV-2 genome (Wuhan-Hu-1) with the ORF of N gene replaced by GFP cDNA, were amplified by PCR *in vitro*. During the PCR reaction, the T7 promoter was introduced to the 5ʹ end of the first fragment, and the designed restriction endonuclease sites (BsaI or BsmBI) were introduced to the corresponding ends of PCR products, which were then used to ligate the neighboring DNA fragments in the right order. The *in vitro* ligated full-length SARS-CoV-2 GFP/ΔN DNA was used as the template to transcribe the viral RNA with mMESSAGE mMACHINE T7 Transcription Kit (Thermo Fisher; AM1344). The mixture of 20 μg of viral RNA and 20 mg N mRNA was added to a 4-mm cuvette containing 0.4 mL of Caco-2-N cells (83×10^6^) in Opti-MEM. A single electrical pulse was given with a GenePulser apparatus (Bio-Rad) with setting of 270 V at 950 μF. Three days post electroporation, P0 virus-like-particles were collected. The expression of N-WT or N-F17A in Caco2 cells was accomplished by lentiviral transduction with pLVX plasmid as the transfer vector. Both Caco2-N (WT) and N (F17A) cells were infected with trVLPs at an MOI of 0.05 for 36 h. The newly reproduced trVLPs in the cell culture supernatant were collected and used to infect fresh Caco-2-N cells. The percentage of GFP-positive cells at 36 hpi was determined by FACS.

#### SARS-CoV-2 mutant generation

The WT SARS-CoV-2 sequence was based on the USA-WA1/2020 strain. It was distributed by the World Reference Center for Emerging Viruses and Arboviruses (WRCEVA) and originally isolated by the USA Centers for Disease Control and Prevention.^75^ Recombinant mutant and WT SARS-CoV-2 were created using the cDNA clone described previously.^73,106^ The generation of recombinant SARS-CoV-2 viruses was approved by the University of Texas Medical Branch Biosafety Committee.

#### SARS-CoV-2 *in vitro* infection

Infection of Vero E6 and Calu-3 2b4 cells were performed according to previously published standard protocols.^106,107^ Briefly, cells were infected at a multiplicity of infection (MOI) of 0.01 by removing the growth media and adding virus diluted in PBS. Cells were subsequently incubated for 45 min at 37°C and 5% CO_2_. Cells were then washed three times with PBS and fresh growth media returned. Virus containing growth media was then sampled at the indicated time points, replacing the removed sample with an equal amount of fresh growth media to maintain a consistent total volume. All infections occurred at Biosafety Level 3 (BSL3) facilities at the University of Texas Medical Branch or St. Jude Children’s Research Hospital.

#### Focus forming assay

For viral titrations, focus forming assays were performed as described previously.^108,109^ Briefly, samples (cell supernatants, nasal washes, or homogenized lung tissue) underwent 10-fold serial dilutions and were then used to infect Vero E6 cells seeded to 96-well plates. After a 45-min incubation at 37°C and 5% CO_2_, 100 μL 0.85% methylcellulose overlay was added. After an additional 24-h incubation, methylcellulose was removed by washing three times with PBS and cells were fixed for 30 min in 10% formalin to inactivate SARS-CoV-2. Cells were then removed from the BSL3 and permeabilized with 0.1% saponin/0.1% bovine serum albumin in PBS. Cells were then stained with an anti-SARS-CoV-1/2 nucleocapsid primary antibody (Cell Signaling Technology; 68344) followed an Alexa Flour 555 fluorescent anti-mouse secondary antibody (Invitrogen; A28180). Each well was then imaged with a Cytation 7 cell imaging multi-mode reader (BioTek) and foci counted manually.

#### Immunofluorescence and microscopy

Cells were seeded on the coverslips pre-coated with collagen (Advanced Biomatrix, 5005) in a 24-well plate. For antibody staining, fixed cells were permeabilized with 0.2% Triton X-100 in PBS for 10 min, and blocked with 1% BSA for 1 h. Samples were further incubated with primary antibodies, including antibodies anti-eIF3η (goat, Santa Cruz, sc-16377), anti-G3BP1 (rabbit; Proteintech; 13057–2-AP) and anti-N (mouse; Sino Biological; 40143-MM05), overnight at 4°C, then washed 3 times with PBS and incubated with host-specific Alexa Fluor 488/555/647 (Thermo Fisher) secondary antibodies for 1 h at room temperature. Nuclei were stained with DAPI in PBS (1:10,000, Biotium, 40009). Cell boundaries were stained with Phalloidin cf.532 in PBS (1:500, Biotium, 00051). Samples were mounted in ProLong Glass Antifade mounting medium (Thermo Fisher; P36980). Images were captured using a Leica TCS SP8 STED 3X confocal microscope with a 63x oil objective.

For co-visualizing the SARS-CoV-2 genomic RNA together with proteins, FISH staining was performed before the antibody staining. The FISH protocol was adopted from Rensen et al.^86^ with modifications. In brief, a mixture of SARS-CoV-2 ORF1ab targeting primary probes with the FLAP sequences attached were prehybridized with Cy5-labeled secondary probes with a complementary sequence to FLAP. The hybridization reaction contained 40 pmol of primary probes and 50 pmol of secondary probes in 1X NEBuffer buffer (NEB; B7003S), and was carried out in a PCR machine with the cycles of 85°C for 3 min, 65°C for 3 min, and 25°C for 5 min. Fixed cells were washed twice with wash buffer A (2X SSC, Ambion; AM9770) for 5 min, followed by two 5-min washing steps with washing buffer B (2X SSC and 10% formamide, Ambion; AM9342). In a humidified chamber, cells were placed on parafilm and incubated at 37°C overnight with 2 μL prehybridized FISH probes diluted in 100 μL of hybridization buffer, which contained 10% dextran (Sigma-Aldrich; D8906), 10% formamide, and 2X SSC. Cells were washed in the dark at 37°C for 1 h twice with prewarmed washing buffer B. After two PBS washes, stained cells were fixed with 4% paraformaldehyde in PBS followed by antibody staining as described above.

#### SG assembly using time-lapse live cell microscopy

A Yokogawa CSU W1 spinning disk with Nikon Elements software was used in time-lapse live cell imaging. Imaging was taken using a 603 Plan Apo 1.4NA oil objective and Perfect Focus 2.0 (Nikon) was engaged for the duration of the capture. During imaging, cells were maintained at 37°C and supplied with 5% CO_2_ using a Bold Line Cage Incubator (Okolabs) and an objective heater (Bioptechs). SG formation in cells was monitored for 60 min after the addition of 500 μM sodium arsenite.

#### Enrichment of G3BP1 in SGs

The enrichment percentage of G3BP1 protein in SGs was quantified by CellProfiler as previously described^110^ with minor modifications. Briefly, the boundaries of the eIF3η -positive puncta and the whole cell were identified using CellProfiler. The ratios of G3BP1 antibody staining signal intensity in the eIF3η -positive puncta to that in the entire cell were used as indicators for the enrichment percentages of G3BP1.

### QUANTIFICATION AND STATISTICAL ANALYSIS

One-way ANOVAs and two-tailed Student’s t-test were calculated in GraphPad Prism. Data distribution was assumed to be normal, but this was not formally tested. Graphs for the analysis were made in Microsoft Excel and GraphPad Prism. All errors corresponding to the standard deviation or standard error of the population are described in figure legends. Statistically significant differences and the number of samples analyzed for each experiment are indicated in figure legends.

## Supplementary Material

1

2

3

4

## Figures and Tables

**KEY RESOURCES TABLE:** 

REAGENT or RESOURCE	SOURCE	IDENTIFIER

Antibodies		

anti-G3BP1	BD Biosciences	Cat# 611127, RRID: AB_398438
anti-G3BP1	Proteintech	Cat# 13057-2-AP, RRID: AB_2232034
anti-G3BP2	Thermo Fisher Scientific	Cat# PA5-53776, RRID: AB_2641801
anti-N	Sino Biological	Cat# 40143-MM05, RRID: AB_2827977
anti-N	Sino Biological	Cat# 40143-R019, RRID: AB_2827973
anti-USP10	Proteintech	Cat# 19374-1-AP, RRID: AB_10858617
anti-caprin 1	Proteintech	Cat# 15112-1-AP, RRID:AB_2070016
anti-GST	BioVision	Cat# 3997-100, RRID: AB_222278
anti-FLAG M2 antibody	Sigma-Aldrich	Cat# F1804, RRID:AB_262044
anti-mCherry	BioVision	Cat# 5993-100, RRID: AB_1975001
anti-Hisx6 tag	Abcam	Cat# ab18184, RRID: AB_444306
anti-GFP	Cell Signaling Technology	Cat# 2555, RRID: AB_10692764
anti-eIF3η	Santa Cruz	Cat# sc-16377; RRID: AB_671941
IRDye 800CW Goat anti-Rabbit IgG (H + L)	LI-COR	Cat# 926–32211; RRID: AB_621843
IRDye 680RD Goat anti-Rabbit IgG (H + L)	LI-COR	Cat# 926–68071; RRID: AB_10956166
IRDye 800CW Goat anti-Mouse IgG (H + L)	LI-COR	Cat# 926–32210; RRID: AB_621842
IRDye 680RD Goat anti-Mouse IgG (H + L)	LI-COR	Cat# 926–68070; RRID: AB_10956588
Donkey anti-Rabbit IgG (H + L) Secondary Antibody, Alexa Fluor 555	Thermo Fisher Scientific	Cat# A-31572; RRID: AB_162543
Donkey anti-Goat IgG (H + L) Secondary Antibody, Alexa Fluor 555	Thermo Fisher Scientific	Cat# A-21432; RRID: AB_2535853
Donkey anti-Goat IgG (H + L) Secondary Antibody, Alexa Fluor 647	Thermo Fisher Scientific	Cat# A-21447; RRID: AB_141844
Donkey anti-Mouse IgG (H + L) Secondary Antibody, Alexa Fluor 488	Thermo Fisher Scientific	Cat# A-21202; RRID: AB_141607
Goat anti-Rabbit IgG (H + L) Secondary Antibody, Alexa Fluor 488	Thermo Fisher Scientific	Cat# A-11008; RRID: AB_143165
Goat anti-Mouse IgG (H + L) Secondary Antibody, Alexa Fluor 488	Thermo Fisher Scientific	Cat# A-11029; RRID: AB_138404
Goat anti-Rabbit IgG (H + L) Secondary Antibody, Alexa Fluor 555	Thermo Fisher Scientific	Cat# A-21428; RRID: AB_141784
Goat anti-Mouse IgG (H + L) Secondary Antibody, Alexa Fluor 555	Thermo Fisher Scientific	Cat# A-21422; RRID: AB_141822

Bacterial and virus strains		

Rosetta 2(DE3) Competent Cells	Millipore	Cat# 71400-4
One ShotTOP10 Chemically Competent E. coli	Thermo Fisher Scientific	Cat# C404003
SARS-CoV-2 USA-WA1/2020 strain (WT)	WRCEVA Xie et al., 2020, ref. 73	N/A
SARS-CoV-2 USA-WA1/2020 strain (N-F17A)	This paper	N/A

Biological samples		

SARS-CoV-2 GFP/ΔN transcription- and replication-competent virus-like particle	Ju et al., 2021, ref. 72	N/A

Chemicals, peptides, and recombinant proteins		

N-WT (1–25)	This paper	N/A
ND3L (1–25)	This paper	N/A
N-P13L (1–25)	This paper	N/A
N-F17A (1–25)	This paper	N/A
caprin 1 (361–381)	This paper	N/A
USP10 (2–25)	This paper	N/A
SFV nsP3 (449–473)	This paper	N/A
Lipofectamine 2000	Invitrogen	Cat# 1168019
Protease inhibitor	Roche	Cat# 11697498001
GFP-Trap beads	ChromoTek	Cat# gtma-20
NuPAGE LDS sample buffer (4X)	Thermo Fisher Scientific	Cat# NP0007
20% paraformaldehyde	Electron Microscopy Science	Cat# 15713-S
ProLong Gold Antifade Mountant with DAPI	Thermo Fisher Scientific	Cat# P36931
RIPA Lysis and Extraction Buffer	Thermo Fisher Scientific	Cat# 89900
SimplyBlue SafeStain	Thermo Fisher Scientific	Cat# LC6065
SYBR Gold Nucleic Acid Gel Stain	Thermo Fisher Scientific	Cat# S11494
RNaseA	Thermo Fisher Scientific	Cat# EN0531
Sodium Arsenite Solution	Sigma	Cat# 35000-1L-R
Ficoll400	Sigma	Cat# F2637
Ni-NTA agarose	GE	Cat# 17-5318-02
Glutathione Sepharose 4B	GE	Cat# 17075601
EZ view Red Anti-Flag M2 affinity gel	Millipore Sigma	Cat# F2426
Glutathione reduced	Sigma	Cat# G4251
Imidazole	Sigma	Cat# I2399
(Isopropyl β-D-1-thiogalactopyranoside) IPTG	Goldbio	Cat# 12481C100
Dithiothreitol (DTT)	Sigma	Cat# 43815

Critical commercial assays		

HiScribe high yield RNA synthesis kit	New England Biolab	Cat# E2040S
RNA 3′ end biotinylation kit	Thermo Fisher Scientific	Cat# 20160
NEBuilder HiFi DNA Assembly Master Mix	New England Biolabs	Cat# E2621S
Q5 Site-Directed Mutagenesis Kit	New England Biolabs	Cat# E0554S
mMESSAGE mMACHINE T7 Transcription Kit	Thermo Fisher Scientific	Cat# AM1344

Deposited data		

crystal structure of NTF2L in complex with caprin1 (360–381)	RCSB Protein DataBank (this paper)	PDB: 8TH7
crystal structure of NTF2L in complex with USP10 (2–25)	RCSB Protein DataBank (this paper)	PDB: 8TH6
crystal structure of NTF2L in complex with ND3L (1–25)	RCSB Protein DataBank (this paper)	PDB: 8TH1
crystal structure of NTF2L in complex with N-P13L (1–25)	RCSB Protein DataBank (this paper)	PDB: 8TH5

Experimental models: Cell lines		

Human: HEK293T	ATCC	RRID: CVCL_0063
Human: U2OS	ATCC	RRID: CVCL_0042
Human: Calu3-2b4	ATCC	RRID: CVCL_YZ47
Chlorocebus sabaeus: Vero E6	ATCC	RRID: CVCL_0574
Chlorocebus sabaeus: Vero E6-TMSSPR2	XenoTech	Cat# JCRB1819 RRID: CVCL_YQ49

Experimental models: Organisms/strains		

Golden Syrian hamsters (HsdHan:AURA strain)	Envigo	N/A

Oligonucleotides		

FISH Probes for SARS-CoV-2 gRNA, ref. 86	Integrated DNA Technologies	N/A

Recombinant DNA		

SARS-CoV-2-N-FLAG	This paper	N/A
SARS-CoV-2-N-F17A-FLAG	This paper	N/A
SARS-CoV-2-N-F17W-FLAG	This paper	N/A
SARS-CoV-2-N-(48–419)-FLAG	This paper	N/A
SARS-CoV-2-N-(175–419)-FLAG	This paper	N/A
SARS-CoV-2-N-(210–419)-FLAG	This paper	N/A
SARS-CoV-2-N-(1–361)-FLAG	This paper	N/A
SARS-CoV-2-N-(1–267)-FLAG	This paper	N/A
SARS-CoV-2-N-(1–209)-FLAG	This paper	N/A
pETite-HisSUMO-N	This paper	N/A
pETite-HisSUMO-N-F17A	This paper	N/A
pAcGFP-N	This paper	N/A
pAcGFP-N-F17A	This paper	N/A
pAcGFP-N (1–47)	This paper	N/A
pAcGFP-N (1–35)	This paper	N/A
pAcGFP-N (1–25)	This paper	N/A
EGFP-N-25mer (1–25),	This paper	N/A
EGFP-N-25mer (1–25)-F17A,	This paper	N/A
EGFP-N-25mer (1–25)-D3A,	This paper	N/A
EGFP-N-25mer (1–25)-D3L,	This paper	N/A
EGFP-N-25mer (1–25)-Q9A,	This paper	N/A
EGFP-N-25mer (1–25)-Q9L,	This paper	N/A
EGFP-N-25mer (1–25)-P13A,	This paper	N/A
EGFP-N-25mer (1–25)-P13L,	This paper	N/A
EGFP-caprin1-21mer (361–381),	This paper	N/A
EGFP-caprin1-21mer (361–381)-F372A,	This paper	N/A
EGFP-USP10-25mer (1–25),	This paper	N/A
EGFP-USP10-25mer (1–25)-F10A,	This paper	N/A
EGFP-nsP3-25mer (449–473)	This paper	N/A
EGFP-nsP3-25mer (449–473)-F451/468A	This paper	N/A
mCherry-nsP3-25mer (449–472)	This paper	N/A
G3BP1-EGFP	This paper	N/A
G3BP1-V11A-EGFP	This paper	N/A
G3BP1-F15A-EGFP	This paper	N/A
G3BP1-F15W-EGFP	This paper	N/A
G3BP1-F33A-EGFP	This paper	N/A
G3BP1-F33W-EGFP	This paper	N/A
G3BP1-F112A-EGFP	This paper	N/A
G3BP1-F124A-EGFP	This paper	N/A
G3BP1-F124W-EGFP	This paper	N/A
GST-G3BP1	Ref. 20	N/A
GST-G3BP1-NTF2L	Ref. 20	N/A
GST-G3BP1-deltaNTF2L	Ref. 20	N/A
GST-NTF2L (F33W)	Ref. 20	N/A

Software and algorithms		

ImageJ	NIH	https://imagej.nih.gov/ij/
Image Studio	LI-COR	https://www.licor.com/bio/products/software/image_studio_lite/
GraphPad Prism Software	GraphPad	https://www.graphpad.com/scientificsoftware/prism/
CellProfiler	Broad Institute	https://cellprofiler.org/
Cytoscape version 3.7.1	Cytoscape Consortium	https://cytoscape.org/release_notes_3_7_1.html
Scrubber 2	BioLogic Software	http://www.biologic.com.au/index.html
Pymol	Schrodinger, USA.	https://pymol.org/2/
Phaser	McCoy et al. ref. 97	http://www.phaser.cimr.cam.ac.uk/index.php/Phaser_Crystallographic_Software
Coot	Emsley et al. ref. 98	https://www2.mrc-lmb.cam.ac.uk/personal/pemsley/coot/
Refmac	Vagin et al. Ref. 99	https://www2.mrc-lmb.cam.ac.uk/groups/murshudov/content/refmac/refmac.html
Biorender	Biorender	https://www.biorender.com/
Adobe Illustrator	Adobe	N/A

## Highlights

- Structural and biochemical analyses define the basis of SARS-CoV-2 N interaction with G3BP1
- F17A mutation of N protein specifically abolishes interaction with G3BP1/2
- N-F17A attenuates SARS-CoV-2 replication and pathogenesis *in vivo*
- G3BP1-N interaction suppresses G3BP1-dependent condensation of viral genomic RNA
