# Supplementary material for: Interaction between host G3BP and viral nucleocapsid protein regulates SARS-CoV-2 replication and pathogenicity
Source: Cell Rep. Author manuscript; Available in PMC 2024 Apr 25. (PMC11044841; doi:10.1016/j.celrep.2024.113965)
Supplement: 1 [file NIHMS1980975-supplement-1.pdf]

Cell Reports, Volume *43*

Supplemental information

Interaction between host G3BP and viral nucleocapsid protein regulates SARS-CoV-2 replication and pathogenicity

Zemin Yang, Bryan A. Johnson, Victoria A. Meliopoulos, Xiaohui Ju, Peipei Zhang, Michael P. Hughes, Jinjun Wu, Kaitlin P. Koreski, Jemma E. Clary, Ti-Cheng Chang, Gang Wu, Jeff Hixon, Jay Duffner, Kathy Wong, Rene Lemieux, Kumari G. Lokugamage, R. Elias Alvarado, Patricia A. Crocquet-Valdes, David H. Walker, Kenneth S. Plante, Jessica A. Plante, Scott C. Weaver, Hong Joo Kim, Rachel Meyers, Stacey Schultz-Cherry, Qiang Ding, Vineet D. Menachery, and J. Paul Taylor

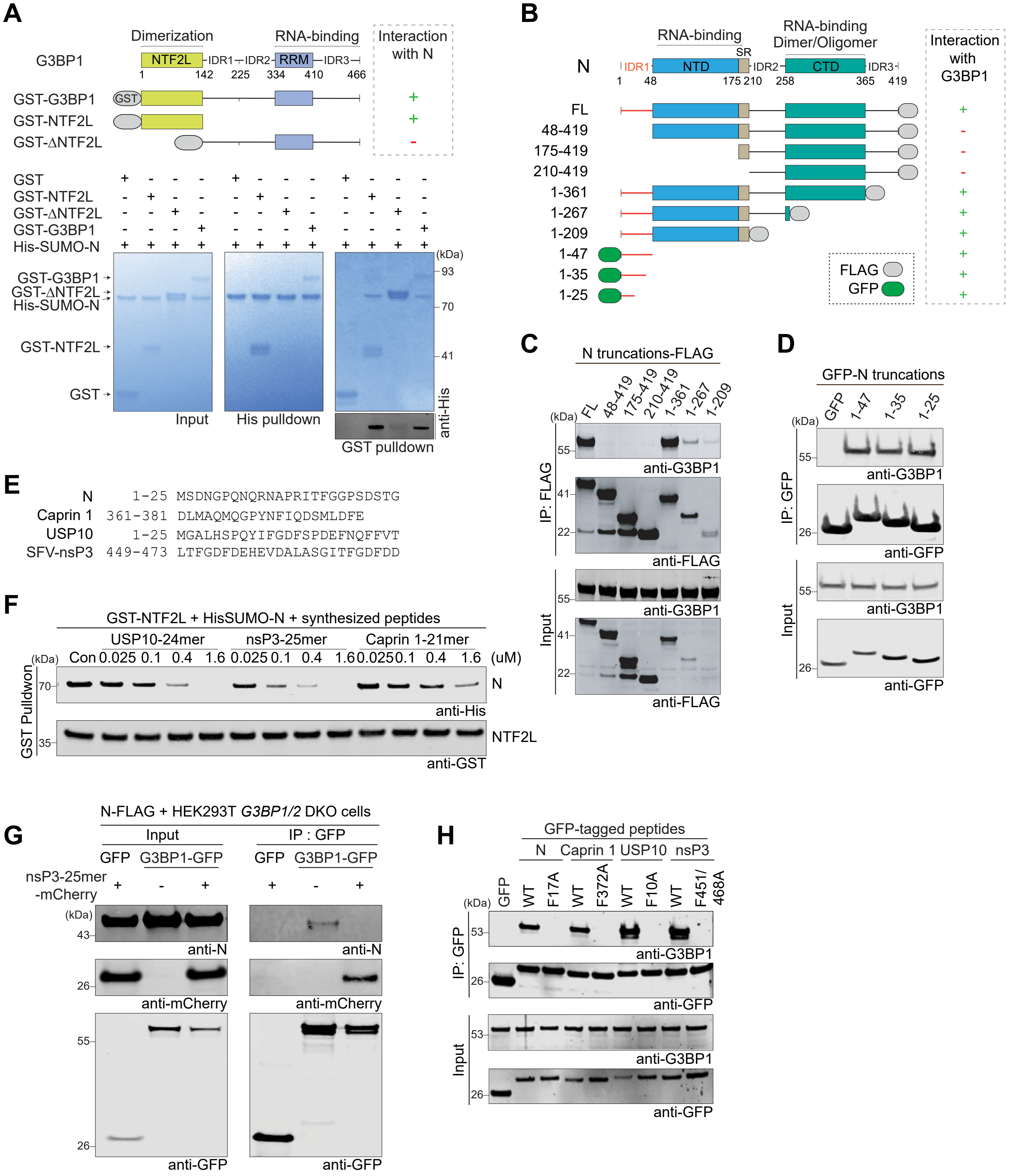
A
Dimerization
RNA-binding
Interaction with N
G3BP1
NTF2L
IDR1
IDR2
RRM
IDR3
1 142 225 334 410 466
GST-G3BP1
GST-NTF2L
GST-ΔNTF2L
GST
+
+
-
GST
GST-NTF2L
GST-ΔNTF2L
GST-G3BP1
His-SUMO-N
(kDa)
93
70
41
GST-G3BP1
GST-ΔNTF2L
His-SUMO-N
GST-NTF2L
GST
Input
His pulldown
GST pulldown
anti-His
B
RNA-binding
RNA-binding Dimer/Oligomer
Interaction with G3BP1
N
IDR1
NTD
SR
IDR2
CTD
IDR3
1 48 175 210 258 365 419
FL
48-419
175-419
210-419
1-361
1-267
1-209
1-47
1-35
1-25
FLAG
GFP
C
N truncations-FLAG
(kDa)
FL
48-419
175-419
210-419
1-361
1-267
1-209
55
41
22
anti-G3BP1
anti-FLAG
IP: FLAG
Input
D
GFP-N truncations
(kDa)
GFP
1-47
1-35
1-25
55
26
anti-G3BP1
anti-GFP
IP: GFP
Input
E
N 1-25 MSDNGPQNQRNAPRITFGGPSDSTG
Caprin 1 361-381 DLMAQMQGPYNFIQDSMLDFE
USP10 1-25 MGALHSPQYIFGDFSPDEFNQFFVT
SFV-nsP3 449-473 LTFGDFDEHEVDALASGITFGDFDD
F
GST-NTF2L + HisSUMO-N + synthesized peptides
USP10-24mer
nsP3-25mer
Caprin 1-21mer
(kDa)
Con
0.025 0.1 0.4 1.6
0.025 0.1 0.4 1.6
0.025 0.1 0.4 1.6
(uM)
70
35
N
NTF2L
anti-His
anti-GST
GST Pulldown
G
N-FLAG + HEK293T G3BP1/2 DKO cells
Input
IP : GFP
GFP
G3BP1-GFP
nsP3-25mer -mCherry
(kDa)
43
26
55
26
anti-N
anti-mCherry
anti-GFP
H
GFP-tagged peptides
N
Caprin 1
USP10
nsP3
(kDa)
GFP
WT
F17A
WT
F372A
WT
F10A
WT
F451/468A
53
26
anti-G3BP1
anti-GFP
IP: GFP
Input

Figure S1. N_{1-25} directly interacts with G3BP1 NTF2L domain and competes with other NTF2L interactors. Related to Figure 1.
(A) Top, domain organization of G3PB1 proteins. Bottom, *in vitro* pulldown assays with purified His-SUMO-N protein and indicated GST-G3BP1 proteins (full-length, NTF2L, ΔNTF2L). Proteins were visualized by Coomassie blue staining (blue gels) and anti-His immunoblot (grayscale image).
(B) N protein truncations used to assess G3BP1 binding in (C) and (D).
(C) HEK293T cells were transfected with indicated FLAG-tagged N protein truncations. Cell extracts were captured with magnetic beads conjugated with FLAG antibody for IP and bound proteins were analyzed by immunoblot.
(D) HEK293T cells were transfected with GFP-tagged N protein fragments. Cell extracts were captured with magnetic beads conjugated with GFP antibody for IP and bound proteins were analyzed by immunoblot.
(E) Amino acid sequences of synthesized G3BP-interacting peptides derived from N, caprin 1, USP10, and SFV-nsP3.
(F) GST pulldown assay with purified GST-NTF2L (100 nM) and His-SUMO-N (100 nM) with increasing concentrations of caprin 1, USP10, or nsP3 peptides.
(G) HEK293T *G3BP1/2* DKO cells were transfected with indicated constructs. Cell extracts were captured with magnetic beads conjugated with GFP antibody for IP and bound proteins were analyzed by immunoblot.
(H) HEK293T cells were transfected with GFP-tagged NTF2L-interacting peptides. Cell extracts were captured with magnetic beads conjugated with GFP antibody for IP and bound proteins were analyzed by immunoblot.

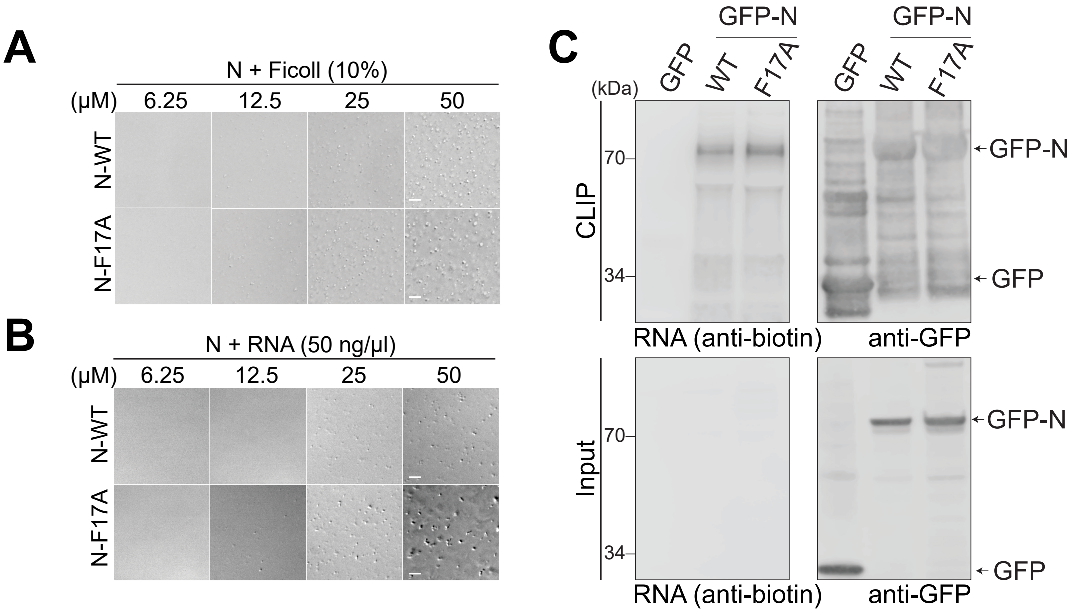

Figure S2. N-F17A does not alter the *in vitro* phase separation behavior and RNA binding of N protein. Related to Figure 1.

(A-B) LLPS of purified N-WT and N-F17A with Ficoll (10%) (A) or 50 ng/μl RNA (B) from HEK293T cells. Scale bar, 50 μm.

(C) CLIP analysis of GFP-N-WT and GFP-N-F17A transiently expressed in HEK293T cells. Immunoprecipitated N protein cross-linked to RNA was assessed by immunoblotting for biotin (RNA) and GFP (N protein).

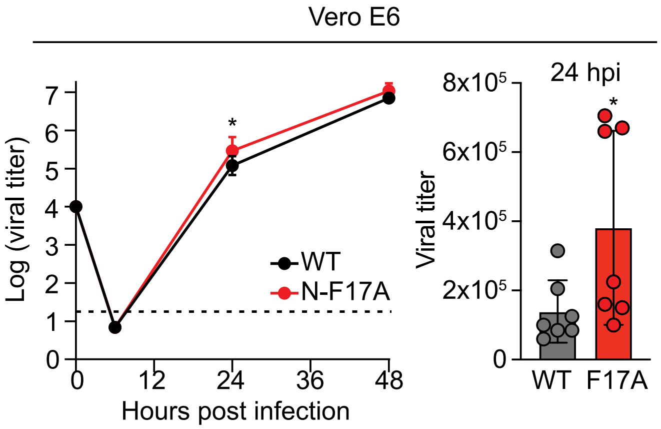

Figure S3. N-F17A increases SARS-CoV-2 replication in Vero E6 cells. Related to Figure 4.
Vero E6 cells were infected with WT or mutant SARS-CoV-2 (N-F17A) at MOI of 0.01. Viral titers were determined at 0, 6, 24 and 48 hpi. Error bars represent mean ± SD. *$p < 0.05$ by two-tailed Student's t-test.

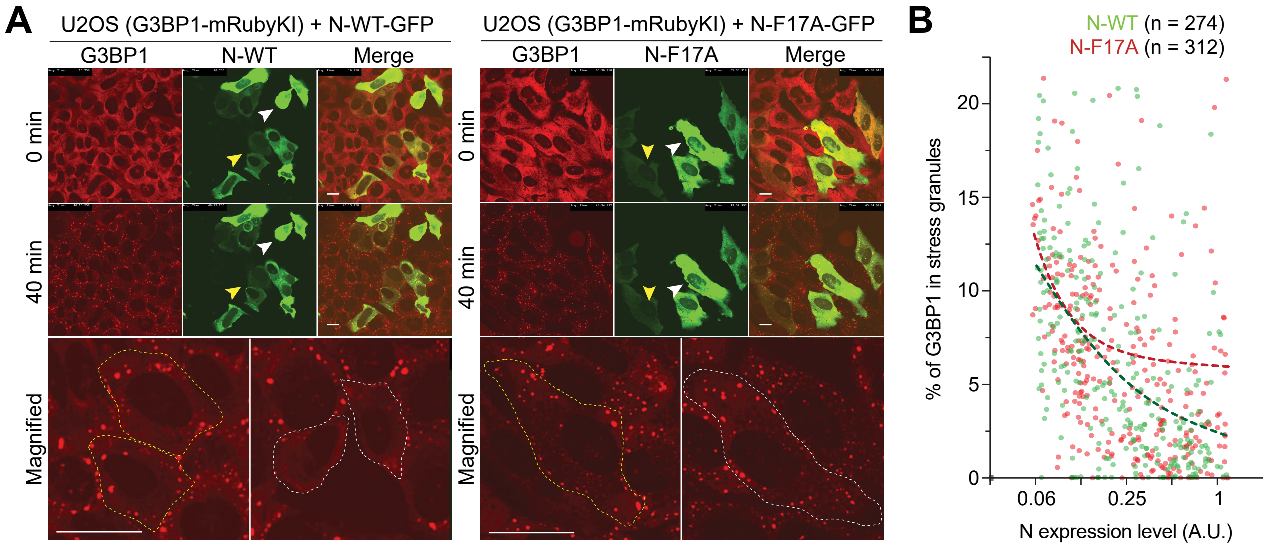

Figure S4. N suppresses SG in an expression level-dependent manner. Related to Figure 6.

(A) G3BP1-tdTomato-KI U2OS cells were transfected with GFP-tagged N-WT or N-F17A. SG formation was induced by 500 µM sodium arsenite and monitored with live cell imaging for 1 h. Representative images are shown at 0- and 40-min post treatment. Yellow and white arrowheads indicate low- and high-expressing cells, respectively, as shown in higher magnification images.
(B) Images of 274 cells transfected with GFP-N-WT (green) and 312 cells transfected with GFP-N-F17A (red) were analyzed quantitatively. N protein expression levels were measured by GFP intensity and SG formation was measured by the enrichment ratio of G3BP1 inside cytoplasmic puncta. The dotted trendlines indicates the inverse correlation between N protein expression level with G3BP1 enrichment inside SGs. Scale bar, 20 µm.

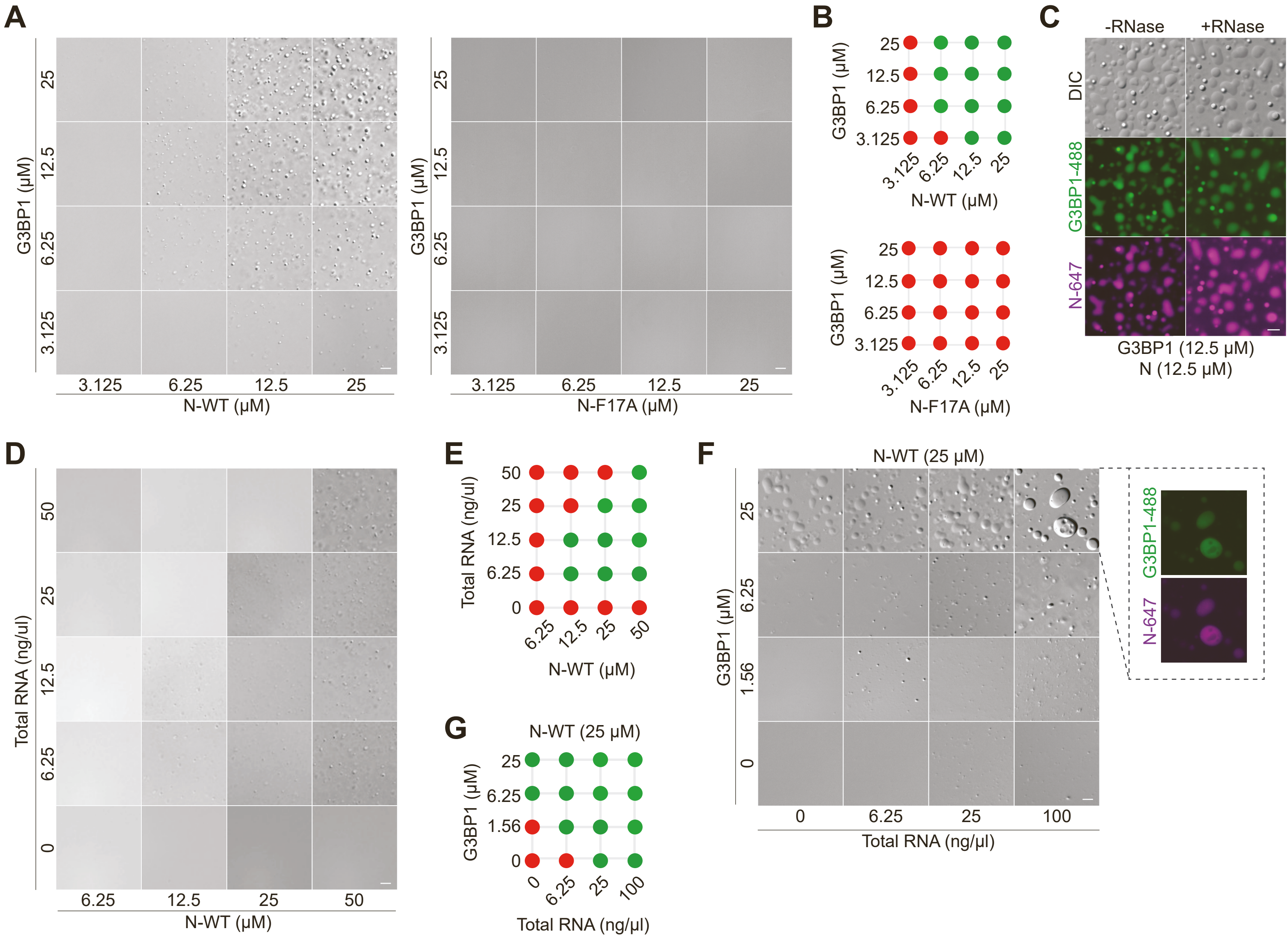

Figure S5. G3BP1 facilitates N protein condensation with RNA. Related to Discussion.
(A-B) N protein does not undergo phase separation alone at physiological salt concentrations (150 mM NaCl). The addition of purified G3BP1 induces co-condensation with N-WT, but not N-F17A. Scale bar, 50 μm.
(C) G3BP1 co-condensation with N protein does not require RNA. Purified G3BP1 and N protein are labeled with Alexa Fluor 488 and Alexa Fluor 647, respectively. 150 mM NaCl, 10 μg/ml RNase A. Scale bar, 50 μm.
(D-E) N protein and RNA undergo co-condensation at physiological salt concentration (150 mM NaCl). Total RNA was purified from HEK293T cells. Scale bar, 50 μm.
(F-G) The addition of purified G3BP1 lowers the concentration threshold for co-condensation of N protein and RNA. Total RNA was purified from HEK293T cells. Scale bar, 50 μm.

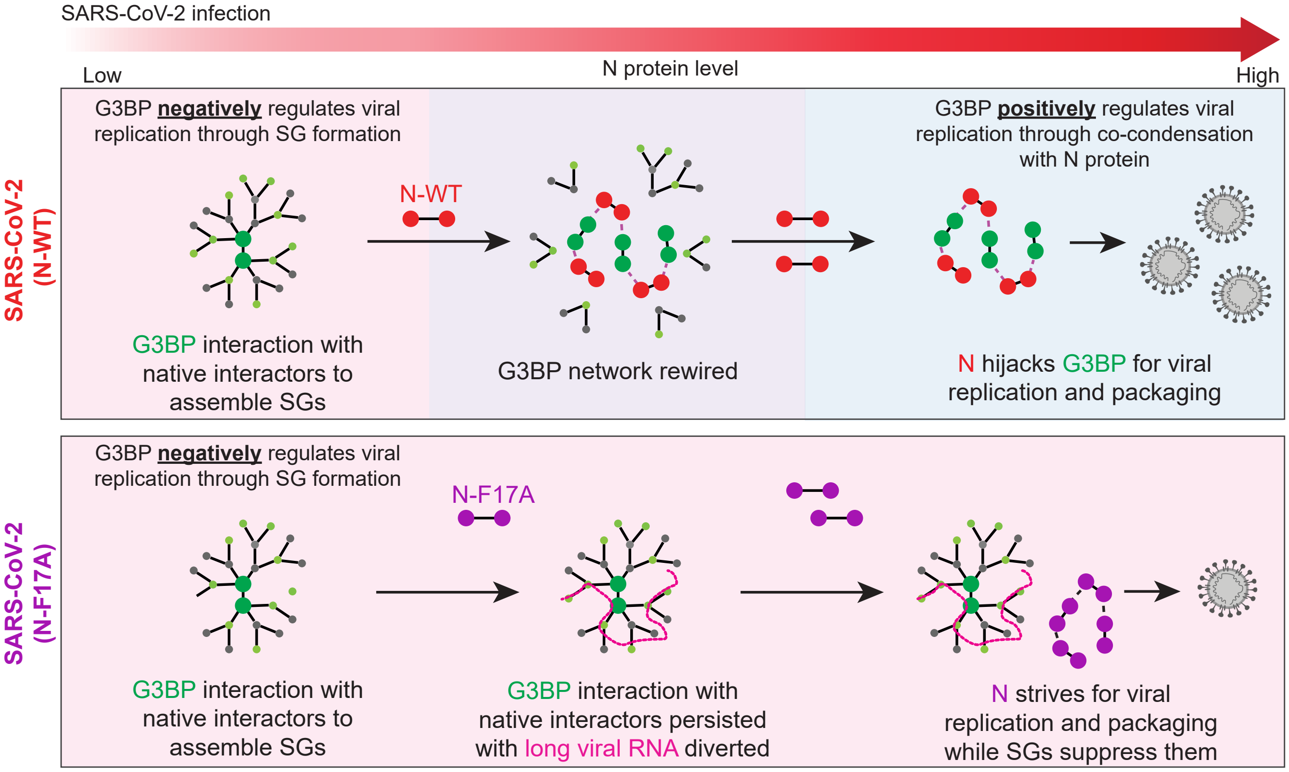

Figure S6. Working model: the role of G3BP-N interaction in SARS-CoV-2 infection. Related to Discussion.
In the early stage of infection, when the expression level of the viral N protein is low, G3BP1/2 predominantly interact with native interactors to assemble the G3BP-centered protein interaction SG core network. This network monitors the rise of cellular RNA levels – a consequence of both host translation shutdown and viral RNA reproduction – by triggering SG formation in infected cells. SGs preferentially sequester long viral RNAs to limit their access to translation and replication machinery, resulting in the suppression of virus replication. During the late infection stage, when the expression level of the N protein is high, this suppression effect is released. N binds to the same pocket on the G3BP-NTF2L domain, outcompeting native G3BP interactors. This rewires the G3BP protein interaction network, leading to SG disassembly and the release of the sequestered viral long RNA. Moreover, beyond SGs, the new G3BP-N interaction network facilitates interaction between N and the viral RNAs. This interaction may further enhance virus replication by promoting vRNA translation, replication, and packaging.

Table S1: Comparative Analysis of Interactomes for GFP, GFP-N, and GFP-N-F17A in HEK293T Cells, and GFP-N in HEK293T *G3BP1/2 DKO* Cells. Related to Figure 1.

Table S2: Comparative Analysis of Interactomes for G3BP1-WT-GFP, G3BP1-V11A-GFP, G3BP1-F33W-GFP, G3BP1-F112A-GFP, and G3BP1-F124W-GFP in HEK293T *G3BP1/2 DKO* Cells. Related to Figure 3.

Table S3. Crystallographic Statistics of G3BP1-NTF2L in Complex with Peptides Derived from N-D3L, N-P13L, caprin1, and USP10. Related to Figure 1.
