# Supplementary material for: Interaction between host G3BP and viral nucleocapsid protein regulates SARS-CoV-2 replication and pathogenicity
Source: Cell Rep. Author manuscript; Available in PMC 2024 Apr 25. (PMC11044841; doi:10.1016/j.celrep.2024.113965)
Supplement: 4 [file NIHMS1980975-supplement-4.docx]

**Table S3. Crystallographic statistics**

|  | G3BP1 NTF2L / Cap1 22mer  (PDB: 8TH7) | G3BP1 NTF2L / SARs N D3L 25mer  (PDB: 8TH1) | G3BP1 NTF2L / SARs N P13L 25mer  (PDB: 8TH5) | G3BP1 NTF2L / USP10 24mer  (PDB: 8TH6) |
| --- | --- | --- | --- | --- |
| **Data collection** |  |  |  |  |
| Space group | P2_1_ | P2_1_ | P1 | P2_1_ |
| Cell dimensions |  |  |  |  |
| a, b, c (Å) | 42.65, 71.44, 51.95 | 52.88, 85.89, 70.69 | 61.6, 69.45, 101.04 | 58.39, 78.38, 73.41 |
| α, β, γ (°) | 90, 111.26, 90 | 90, 110.5, 90 | 70.657, 76.857, 88.305 | 90, 108.63, 90 |
| Resolution (Å) | 48.42 – 2.88  (3.04 – 2.88)^a^ | 66.22 – 1.80 (1.84 – 1.80) | 92.77 – 2.623  (2.74 – 2.62) | 45.21 – 2.34  (2.42 – 2.34) |
| R_merge_ | 0.134 (0.554) | 0.221 (1.005) | 0.072 (0.305) | 0.153 (0.360) |
| I/σI | 8.5 (2.3) | 12.9 (2.3) | 10.3 (3.5) | 9.2 (3.3) |
| Completeness (%) | 99.5 (99.7) | 99.1 (99.6) | 71.4 (7.8) | 63.1 (6.8) |
| Redundancy | 4.5 (4.5) | 6.8 (6.8) | 3.5 (3.7) | 3.3 (3.4) |
| **Refinement** |  |  |  |  |
| No. reflections | 6159 | 51633 | 31252 | 15966 |
| R_work_/R_free_ | 0.190 / 0.277 | 0.200 / 0.238 | 0.231 / 0.338 | 0.217 / 0.295 |
| No. atoms |  |  |  |  |
| Protein | 2405 | 4586 | 10321 | 4868 |
| Water | 12 | 284 | 64 | 68 |
| Wilson B-factor (Å^2^) | 44.6 | 26.1 | 38.0 | 29.9 |
| r.ms.d. |  |  |  |  |
| Bond lengths (Å) | 0.007 | 0.010 | 0.006 | 0.005 |
| Bond angles (º) | 1.600 | 1.608 | 1.506 | 1.215 |
| Ramachandran statistics (%) |  |  |  |  |
| Favored regions | 87.29 | 95.77 | 86.36 | 91.54 |
| Outliers | 3.01 | 0.74 | 3.83 | 1.21 |

^a^ = highest resolution shell in parenthesis
